# Lack of adducin impairs the stability of endothelial adherens and tight junctions and may be required for cAMP-Rac1-mediated endothelial barrier stabilization

**DOI:** 10.1038/s41598-022-18964-5

**Published:** 2022-09-02

**Authors:** Sina Moztarzadeh, Mariya Y. Radeva, Sara Sepic, Katharina Schuster, Ibrahim Hamad, Jens Waschke, Alexander García-Ponce

**Affiliations:** Chair of Vegetative Anatomy, Faculty of Medicine, Ludwig-Maximilians-University (LMU) Munich, Pettenkoferstraße 11, 80336 Munich, Germany

**Keywords:** RHO signalling, Adherens junctions, Tight junctions

## Abstract

Adducin (Add) is an actin binding protein participating in the stabilization of actin/spectrin networks, epithelial junctional turnover and cardiovascular disorders such as hypertension. Recently, we demonstrated that Add is required for adherens junctions (AJ) integrity. Here we hypothesized that Add regulates tight junctions (TJ) as well and may play a role in cAMP-mediated barrier enhancement. We evaluated the role of Add in MyEnd cells isolated from WT and Add-Knock-Out (KO) mice. Our results indicate that the lack of Add drastically alters the junctional localization and protein levels of major AJ and TJ components, including VE-Cadherin and claudin-5. We also showed that cAMP signaling induced by treatment with forskolin and rolipram (F/R) enhances the barrier integrity of WT but not Add-KO cells. The latter showed no junctional reorganization upon cAMP increase. The absence of Add also led to higher protein levels of the small GTPases Rac1 and RhoA. In vehicle-treated cells the activation level of Rac1 did not differ significantly when WT and Add-KO cells were compared. However, the lack of Add led to increased activity of RhoA. Moreover, F/R treatment triggered Rac1 activation only in WT cells. The function of Rac1 and RhoA per se was unaffected by the total ablation of Add, since direct activation with CN04 was still possible in both cell lines and led to improved endothelial barrier function. In the current study, we demonstrate that Add is required for the maintenance of endothelial barrier by regulating both AJ and TJ. Our data show that Add may act upstream of Rac1 as it is necessary for its activation via cAMP.

## Introduction

The endothelium is a single layer of endothelial cells lining the interior surface of blood vessels, thereby acting as a gatekeeper controlling the passage of water, blood proteins and circulating cells through the vessel walls. This exclusive feature of the endothelium is mediated either by a specific transcellular system of vesicle transport, where the components travel through the endothelial cell’s wall or via paracellular transport, where the blood elements pass in between neighboring endothelial cells by coordinate opening and closure of their cell–cell contacts^1–5^.

To preserve tissue homeostasis and prevent cell damage, tight regulation of endothelial permeability is necessary. Mainly in post-capillary venules, vascular damage and increase junctional leakage is manifested during various malignant and non-malignant diseases such as allergy, arteriosclerosis, diabetes, inflammation and oedema, a pathological process that results in the local release of pro-inflammatory mediators which affect the permeability of adjacent blood vessels^6–9^. All these conditions may lead to severe organ dysfunction or fatal outcome.

Two types of cell–cell contacts, i.e. the adherens junction (AJ) serving mechanical anchorage of opposing cells and the tight junction (TJ) sealing the intercellular cleft between adjacent cells determine endothelial barrier function and are responsible for limiting paracellular permeability^10,11^. VE-cadherin is the critical molecule involved in AJ organization, while claudin-5 and occludin are the key TJ components. Both types of barrier forming junctions are tethered via adaptor molecules (ß-catenin and plakoglobin for AJs as well as ZO-1 for TJs) to the actin cytoskeleton. The latter is an extremely dynamic structural unit associated with the regulation of cell–cell complexes and can be modulated by actin binding proteins (ABPs) such as adducin (Add). Therefore, preservation of the endothelial barrier is not only achieved by controlling the stability of the intercellular junctions but also by remodeling of the actin cytoskeleton^10,12^.

Add is a membrane skeletal protein that binds to the barbed ends of actin filaments, thereby favoring the assembling of F-actin with spectrin to form spectrin-actin meshwork beneath the cell membrane. This spectrin-based network provides a physical support to the plasma membrane and mediates cell signaling in different physiological processes^13–16^. In addition, as an ABP, Add is crucial for the regulation of the filament dynamics by capping the fast-growing ends^15^ and facilitating the bundling of the actin filaments^17^. Because of this, Add promotes cell motility and migration^18,19^. In addition, Add was reported to exert multiple cellular functions in epithelia, playing prominent role in the stabilization of cell–cell adhesion, contact formation and maintenance^20–23^. Similarly to others, our group recently demonstrated that loss of Add leads to reduce intercellular adhesion^24^ and it is required for coordinated assembling of desmosomes, an important type of junctions linking neighboring epithelial- and non-epithelial cells^25^. Furthermore, we have shown that Add is involved in the stabilization of the endothelium by remodeling Ca^2+^dependent endothelial AJs^26^. However, little to nothing is known on how Add correlates with junctional or junctional-related proteins, as well as with crucial signaling molecules involved in modulation of endothelial barrier function under basal state and after cAMP elevation. Therefore, by taking the advantage of the newly established Add-deficient mouse microvascular myocardial endothelial cells (MyEnd) cell line, we provide new insights about the role of Add in regulation and maintenance of basal and cAMP-mediated endothelial barrier stability, which could be potentially exploited as new therapeutic targets to treat endothelial hyperpermeability.

## Material and methods

All experiments reported here were repeated at least 3 times or more. “N” indicates the number of biological independent experiments for each particular analysis.

### Cell culture and endothelial cell isolation ex-vivo

The generation of immortalized mouse microvascular myocardial endothelial cells (MyEnd) was performed similary to previously described methods in the literature^27,28^. All endothelial cell isolations were carried out ex-vivo after mice sacrifice in a S2 cell culture laboratory in our facilities. Mice were handled by Dr. Mariya Y. Radeva, who has been certified with a FELASA-C course for lab animal training in Germany. Briefly, myocardial tissues harvested from WT and Add-KO animals were chopped into small pieces and further processed with a cell-dissociation solution containing trypsin and collagenase A enzymes. The digestion process was terminated by addition of equal volume of ice-cold buffer A (153 mM NaCl, 5.6 mM KCl, 2.3 mM CaCl_2_ × 2H_2_O, 2.6 mM MgCl_2_ × 6H_2_O, 15 mM HEPES, 1% BSA, Sigma Aldrich Chemie GmbH, Taufkirchen, Germany). The resulting cell suspension was shortly centrifuged and the cell pellet was resuspended in Dulbecco’s Modified Eagle’s medium (DMEM, Gibco-ThermoFisher, Munich, Germany, Cat# 41,966-029), supplemented with 50 U/mL Penicillin G/Streptomycin (Sigma Aldrich Chemie GmbH, Taufkirchen, Germany), and 10% Fetal Calf Serum (FCS, Biochrom, Berlin, Germany, Cat# S0115/0247X). The cells were plated onto gelatin-coated culture dishes. To achieve successful immortalization, 24 h later the attached cells were treated with Polyoma virus middle T Antigen, secreted by GP + E-86 Neo fibroblasts for two additional days. Then, the surviving endothelial cells were grown in DMEM medium only with supplements and FCS until confluent monolayer was achieved. To confirm the endothelial phenotype, cells were tested by Western blot and immunostainings for the presence of endothelial cell markers.

### Ethical approval

Experiments were approved by the Ethics committee of the Regierung von Oberbayern, Germany (Az. 55.2-2532.Vet_02-14-139) and all of them were performed in accordance with the relevant guidelines and regulations.

### Antibodies and test reagents

The following antibodies were used in this study: rabbit anti-FoxO1 (*C29H4*), Cat. #2880, Cell Signaling, mouse anti-Plakoglobin, [Clone PG 5.1], Cat# 61,005, from Progen; mouse anti-RhoA, Cat. # ARH04), offered by Cytoskeleton; mouse anti-CD31/PECAM-1 Antibody (H-3), sc*-*37676 from Santa Cruz*;* mouse anti-Rac1, Cat. # 610,651 and mouse anti-β*-*catenin*,* BD 61,054, both bought from BD Transduction Laboratories; rabbit anti-ZO-1, Cat. # 61-7300 and rabbit anti-occludin, Cat. # 40-4700, both obtained from Thermo Fisher Scientific; rabbit anti-claudin 5*,* ab15106, rabbit anti-α-adducin, ab51130*,* rabbit anti-VE-cadherin, ab33168, mouse anti α–tubulin, ab7291 and rabbit anti-VE-cadherin, ab33168, all purchased from Abcam.

Intracellular cAMP elevation was induced by simultaneous administration of 5 µM Forskolin (F) (Sigma Aldrich Chemie GmbH, R6520, Taufkirchen, Germany), an adenylyl cyclase (AC) activator, and 10 µM Rolipram (R), (Sigma Aldrich Chemie GmbH, F6886, Taufkirchen, Germany), a phosphodiesterase 4 (PDE4) inhibitor (Sigma Aldrich Chemie GmbH, R6520, Taufkirchen, Germany) for 1 h. Specific activation of Epac1 was achieved by treating the cells with “007” (Biolog, Cat. No. C051-05, Bremen, Germany) for 1 h at a final concentration of 200 µM. Synchronized, direct activation of Rho family small GTPases (RhoA/Rac1/Cdc42) was induced by application of 0.25 µg/mL Rho/Rac/Cdc42 Activator I (Cytoskeleton Inc, Cat#CN04, Denver, CO, U.S.A.) for 2 h. Indirect activation of RhoA was achieved by addition of Rho Activator I (Calpeptin; Cytoskeleton Inc, Cat#CN01) to a final concentration of 1 unit/mL for 1 h.

### Western blots

MyEnd cells were grown to confluency, washed with ice-cold PBS and subsequently lysed with SDS-lysis buffer (25 mM HEPES, 2 mM EDTA, 25 mM NaF and 1% SDS, pH 7.6), containing cOmplete™ protease inhibitor cocktail (Roche Diagnostics, #11,697,498,001, Mannheim, Germany). After sonication, the protein concentration was estimated with the Pierce BCA protein assay kit (Thermo Fischer Scientific, Waltham, Germany). An equal amount of total protein was loaded on polyacrylamide gels and subsequently, the separation of the proteins based on their molecular weight was done by electrophoresis. Proteins were then transferred to nitrocellulose membranes, blocked with 5% non-fat milk in TBS-Tween (0.1%), and probed overnight at 4 °C with the antibodies of interest. Images were captured using the Amersham Imager 600 (AI600, GE Healthcare, Munich, Germany). Densitometric analysis was done with ImageJ, as described elsewhere^29^.

### Immunoprecipitation

To check whether the protein partners of VE-cadherin- based complex change due to lack of Add, pull down with goat anti-VE-cadherin or mouse-anti-β-catenin antibodies was completed. To further validate the complex between VE-cadherin, ß-catenin and plakoglobin (PG), immunoprecipitation with mouse anti-ß-catenin antibody was performed. In brief, WT and Add KO cells were grown to confluency, washed twice with ice-cold washing buffer containing 0.1% Nonidet-P40 (NP-40) (10 mM HEPES, pH 7.9, 1.5 mM MgCl_2_ × 6H_2_O, 10 mM KCl, 5 mM EDTA, 0,1% NP-40). Then, cells were lysed for 30 min with ice-cold buffer having 10 mM HEPES, pH 7.9, 1,5 mM MgCl_2_ × 6H_2_O, 10 mM KCl, 5 mM EDTA, 2 mM EGTA and 1% NP-40 plus cOmplete™ protease inhibitor cocktail. Lysates were subsequently homogenized by passaging them through a 20G needle 12 times. The samples were then cleaned from cell debris during a 3 min centrifugation at 11 000 rcf. Protein concentration was estimated using the BCA method. An equal amount of protein (600 µg) from each cell type was incubated with pre-cleared A (for VE-Cadherin) or G (for β-catenin) agarose beads for 1.5 h. Thus, the unspecific binding to the beads was reduced. Lysates were incubated overnight with either of the antibodies used for pull-down or with the respective IgG control. The next day, the protein complex bound to the specific antibody was trapped utilizing beads for 2 h at 4 °C. The Beads were then washed and mixed with Laemmli buffer. After denaturation at 95 °C for 10 min, the protein complex was subjected to Western blot analysis.

### Evaluation of the mRNA levels by PCR analysis

Total RNA extracted from WT and Add-KO cells (RNeasy Plus Mini Kit, Cat# 74,134, QIAGEN) was used for cDNA amplification using the SuperScript™ II Reverse Transcriptase (Cat# 18,064,014, Thermo Fisher) according to the manufacturer’s instructions. The cDNA was used as a template for PCR carried out to amplify the desired targets of interest. All primers used in the assay are listed in Table 1. Similar PCR conditions were used for all genes of interest: 3 min at 95 °C, 35 cycles of 30 s at 95 °C, 30 s at 55°/60 °C followed by 45 s at 72 °C. The PCR was completed with a final extension of 10 min at 72 °C. ß2-microglobulin (B2M) was utilized to confirm equal loading on the agarose gel. In addition, B2M primer pair was able to detect any genomic DNA contamination. Moreover, the primer pair for adducin give an amplicon only when cDNA from WT was used. If Add-KO cDNA was tested, no product was visualized, because both primers align to the excised sequence in the Add mutant mouse model used for generation of the MyEnds.

**Table 1:** Primer sequences and amplicon size corresponding to each indicated gene.

Target analyzed	5’- > 3’	Amplicon size (bp)
Adducin	FW: GTACAGCGATGTGGAAGTCC REV: CTGTCCATCATCATCACACCACAC	374
VE-cadherin	FW: GAGTTCACCTTCTGTGAGGAGATG REV: CTTCTGCACCTGCGTGTACAC	329
ß-catenin	FW: GAGGACCTACACTTATG AGAAGC REV: GGCAGTCCATAATGAAG GCG	492
Claudin-5	FW: GATGTCGTGCGTGGTGCAGAGTAC REV: CTTGTCGTAATCGCCATTGGCCGTG	489
Occludin	FW: CCTCCAATGGCAAAGTGAAT REV: CTCCCCACCTGTCGTGTAGT	248
ZO-1	FW: CCACCTCTGTCCAGCTCTTC REV: CACCGGAGTGATGGTTTTCT	248
B2M	FW: CAAGTATACTCACGCCACCCAC REV: CATCATGATGCTTGATCACATGTCTC	292

### Transendothelial electrical resistance (TER) measurements

The function of the endothelial barrier was examined by using the ECIS Z Theta system (Applied Biophysics Inc, Troy, NYC, U.S.A.) as described previously^27,30^. In brief, MyEnd cells were seeded on 0.5% gelatine pre-coated 8W10E gold microarray electrodes (Ibidi, Martinsried, Germany). Prior to the start of the experiment, the medium was exchanged and the electrodes were mounted into the ECIS system’s array holders, where they were pre-stabilized and equilibrated at 37 °C in a humidified atmosphere of 5% CO_2_, for at least 1 h. Data were acquired with a frequency of 4000 Hz.

### Immunofluorescence stainings

MyEnd were seeded on 0.5% gelatine pre-coated 12 mm glass coverslips and grown to confluence. After treatment application, cells were fixed with 4% paraformaldehyde (PFA) for 10 min at room temperature. Cells were then permeabilized with 0.1% Triton-X-100 in PBS for 5 min and immunolabeling of the proteins of interest was carried out following the standard procedures^26,31^. Cell monolayers were photographed with a confocal microscope (Leica SP5, Mannheim and Wetzlar, Germany) equipped with a HCX PL APO Lambda blue 63 × 1.4 oil immersion objective (Leica). The same microscope settings were used for all conditions tested.

### Quantification of junctional fragmentation

Quantification of VE-cadherin, β-catenin, PG, claudin-5 and ZO-1 junctional signals was performed using ImageJ software. First, Z-stacks were generated from confocal images using the “Z-project” function under the Image > Stacks drop-down menu. The projection type used was “Sum Slices” in order to preserve the original unmodified signal. Once the stacks were done, the image threshold (Image > Adjust > Threshold) was adjusted to only include pixels with values higher than that of the background. Pixels that fall below the background value will yield a mean gray value of 0, which is then interpreted as a gap. In order to accurately measure the positive pixels, the junctional signal was linearized and shrank to a thickness of 1 pixel using the built-in plugin “Skeleton > Skeletonize (2D/3D)”. Once the skeleton is processed, a line was manually drawn on each junction using the “Freehand line” tool with a thickness of 3 pixels, to ensure no signal is lost while drawing the line. Next, the pixel values were measured with the “Plot profile” tool (Ctrl + K). To determine the percentage of fragmentation, the total amount of pixels with a value of 0 were divided by the total amount of pixels measured and multiplied by 100. A minimum of 30–35 junctions from 3 different pictures belonging to 3 independent experiments were used for this quantification.

### Plasmid preparation and transfection

A mouse claudin-5 pEGFP-C1 construct was assembled by subcloning a DNA fragment corresponding to the mouse claudin-5 coding sequence into a pEGFP-C1 expression vector. For this purpose, mRNA extracted from WT MyEnd cells was used for generation of complementary DNA (cDNA). The latter was used as a DNA template in a PCR designed to generate a complete coding sequence for mouse claudin-5 with the help of gene specific primers (FW: TATCCGCTCGAGCTATGGGGTCTGCAGCGTTG; REV: TCTGTAGGATCCTTAGACATAGTTCTTCTTGTCGTAATCG), introducing not only particular restriction sites for subsequent cloning procedure, but also, in case of the FW primer, a Kozak consensus sequence for enhanced translation in mammalian cells. For efficient restriction analysis of the PCR product, the PCR amplicon was additionally 3′ adenylated and cloned into pCRTM2.1-TOPO® vector (ThermoFisher Scientific). The target sequence was then extracted from the TOPO-vector by digestion with respective restriction enzymes and subcloned into pEGFP-C1 vector. Thus, the EGFP tag was fused with the N-terminal end of the mouse claudin-5. Verification of the newly constructed vector was done with both restriction and sequencing analyses.

For plasmid transfection, MyEnd cells were seeded on µ-Slide 8 well Ibidi treated chambers (Ibidi GmbH, Gräfelfing, Germany). Cells were cultured in complete growth medium, supplemented with serum and antibiotic until they reached a confluency of 60–70%. 3 h prior to transfection, serum starvation was done with medium containing 1% FCS. The cells were transfected with either the pEGFP-empty or pEGFP-claudin-5-C1 plasmid, using Lipofectamine 3000 transfection reagent (Thermo Fisher Scientific; Waltham, MA USA). Plasmid DNA-lipid complex was prepared in serum-free medium, according to the manufacturer’s instructions. For each well, 250 ng of plasmid DNA was used. The DNA-lipid complex was incubated for 15 min at room temperature and then added to the cells, already substituted with complete growth medium. 48 h after transfection, cells were fixed and stained with DAPI for nucleus visualization. Cells were imaged by confocal microscopy.

### Calcium switch

The calcium switch assay was performed as previously published^26^. Briefly, cells were seeded on ECIS electrodes and grown to confluency with complete culture medium. Prior to the start of the experiment, cells were given medium containing only 5% FCS. The TER was measured for 1 h and subsequently, EGTA was added to a final concentration of 2.5 mM and incubated for 30 min. Afterwards, CaCl_2_ was given to a final concentration of 5 mM and remained in the medium till the end of the experiment. The TER was monitored for at least 2 h after EGTA application. In case of WB or immunostainings, cells were seeded on 6-well or 24-well plates and treated the same as above. Right after, cells were lysed with SDS-lysis buffer or fixed with 4% PFA respectively and processed further.

### Determination of cyclic-AMP concentration

Cellular cAMP levels were quantified using the commercially available cAMP enzyme linked immunosorbent assay (Sigma Aldrich Chemie GmbH, Taufkirchen, Germany) following the manufacturer’s instructions.

### Colorimetric G-LISA Rac1 and RhoA activity measurements

The intracellular concentration of GTP-bound Rac1 and RhoA GTPases was measured by using G-protein ELISA assays (Cytoskeleton, Denver, CO, U.S.A.). Cells were processed according to the manufacturer’s instructions. The final reaction absorbance was measured using a TECAN, Infinite 200 PRO microplate reader (Tecan Deutschland GmbH, Crailsheim, Germany) with a wavelength of 490 nm.

### Statistical analysis

Data is presented as mean ± SEM. Data analysis was carried out with Prism Software version 8 (Graph Pad, San Diego, California, U.S.A.). Student´s T-test was used when only two groups were compared. To evaluate differences between more three or more groups, Two-way Analysis of Variance (ANOVA) followed by Sidak´s Multiple Comparison Test was applied. The results were considered statistically significant at *p* ≤ 0.05.

## Results

### The complete absence of adducin affects the function and composition of endothelial cell–cell junctions

**Figure 1:**
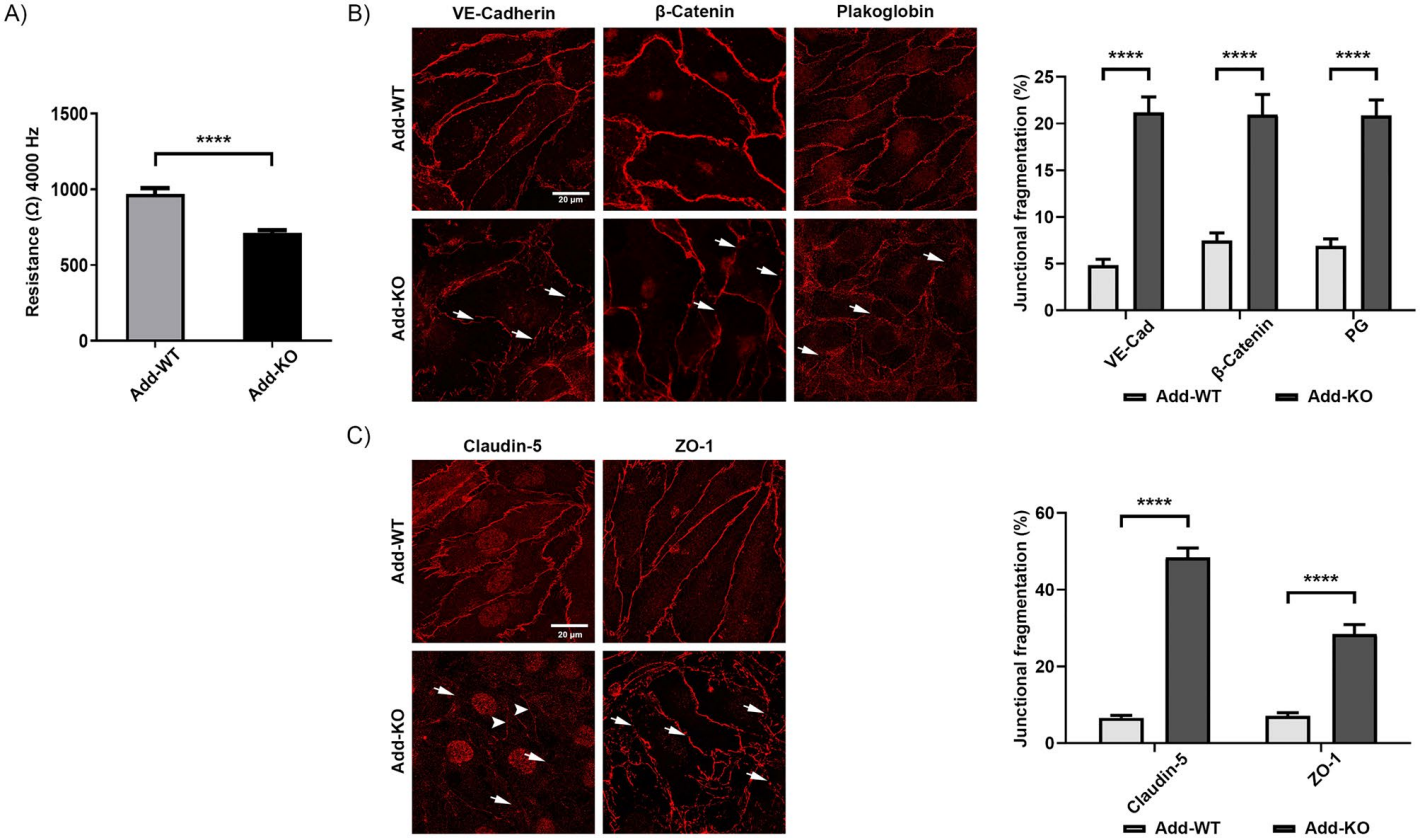
Functional analysis of endothelial barrier and distribution pattern of endothelial AJ and TJ. (**A**) TER measurements of confluent WT and Add-KO cell monolayers under basal conditions (N = 3 per group). Representative immunostainings and corresponding quantification analysis depicting the continuity of the fluorescence intensity at the junctions for (**B**) AJ proteins VE-Cadherin, β-catenin and plakoglobin or (**C**) TJ proteins claudin-5 and ZO-1 from WT and Add-KO cells (N = 3–4 per group). Arrows indicate areas of fragmented membrane staining; arrowheads indicate thin membrane signal. Data are represented as mean ± SEM; ****p < 0.0001.

**Figure 2:**
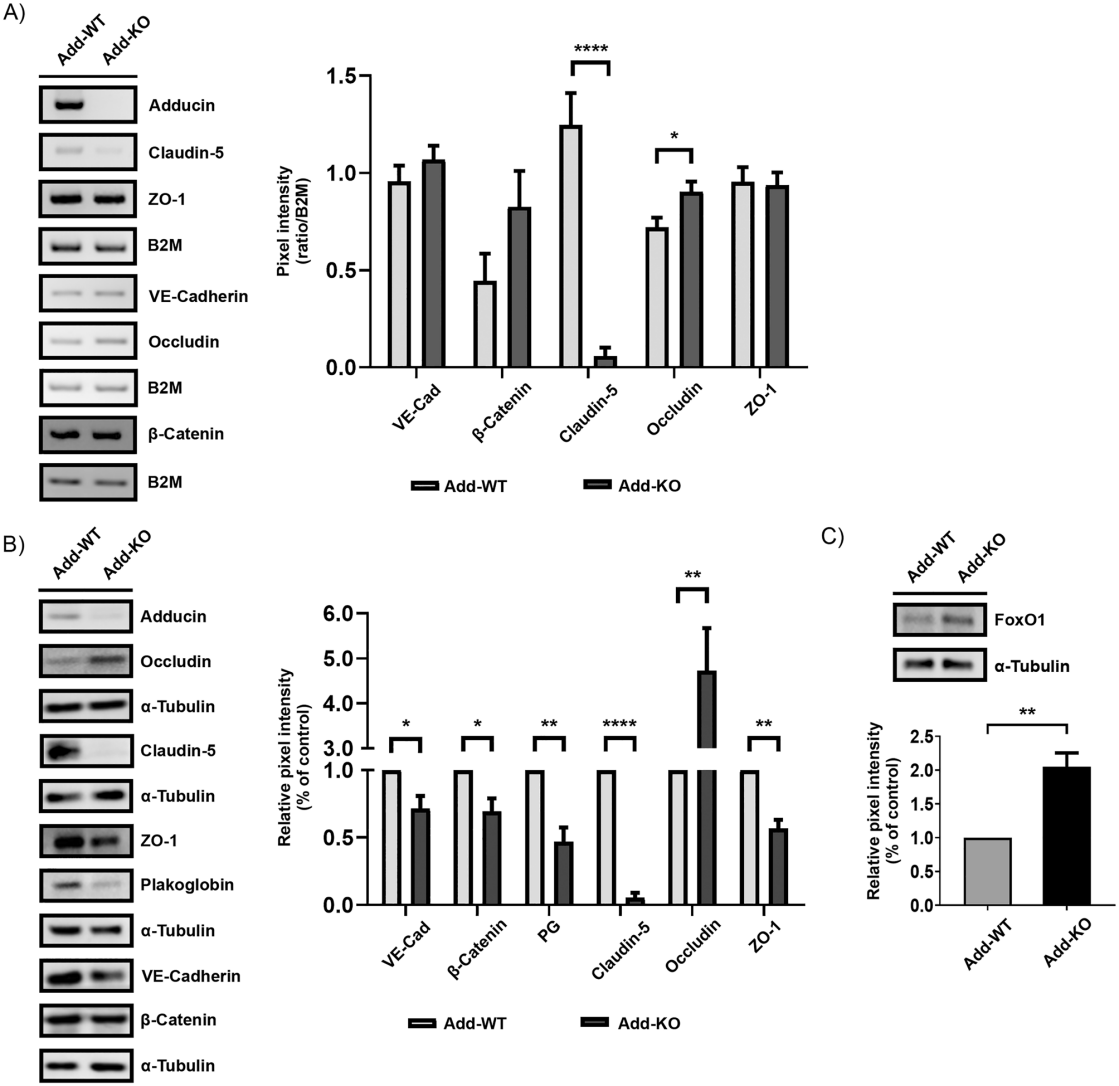
Protein and mRNA expression analysis of junctional and junction-associated molecules. (**A**) Representative RT-PCR for AJ and TJ components from confluent WT and Add-KO monolayers under basal conditions. The graph shows the pixel intensity ratio for each and the corresponding loading control, B2M (N = 8 per group). (**B**) Representative Western blot of junctional and junctional-associated molecules. The graph denotes the ratio between the pixel intensity for each protein and α-tubulin normalized to the respective WT control (N = 5 per group). (**C**) Representative Western blot for the claudin-5 transcriptional repressor FoxO1. The graph presents the relative pixel intensity normalized to the WT controls (N = 4). Data are represented as mean ± SEM; *p < 0.05; **p < 0.01; ****p < 0.0001.

Previously, it was shown that Add may play a critical role in endothelial barrier regulation^26^. In this study, we analyzed this effect in more detail by establishing new myocardial-derived immortalized endothelial cell lines obtained from WT and Add-KO C57BL/6 mice. To test the integrity of the monolayer the TER of confluent MyEnds was analyzed. Similar to our previous observations, Add-KO cell monolayer had significantly lower TER (711.64 ± 92.69 Ω) relative to the Add-WT controls (969.28 ± 179.75 Ω) (Fig. 1A). Next, we investigated if this effect was related to changes in the localization pattern of TJ and AJ components. In the Add-KO cells, the AJ proteins VE-cadherin, β-catenin and plakoglobin showed an overall fragmented membrane staining pattern in contrast to the more defined distribution in WT cells (Fig. 1B, arrows). Similarly, the absence of Add resulted in fragmented ZO-1 localization at the TJ (Fig. 1C, arrows). Furthermore, the distribution of the barrier-sealing claudin-5 at the cell borders was strikingly impaired, leaving areas with big gaps or thin membrane dot-like staining between adjacent cells (Fig. 1C, arrows). The above observations were confirmed by quantification of the junctional fragmentation (Fig. 1B–C). Our data suggest that Add is required for basal barrier function by promoting the proper localization of TJ and AJ molecules. We further investigated whether the changes in the localization pattern of junctional and junctional-related proteins were associated with alterations in their mRNA and protein expression levels, therefore PCR and Western blot analyses were performed. Evaluation of the AJ components showed that protein expression of VE-cadherin, β-catenin and plakoglobin was reduced due to the absence of Add, but no differences at the transcriptional level were detected for VE-cadherin and β-catenin (Fig. 2A,B). Moreover, we found that the lack of Add resulted in significantly diminished mRNA and protein levels of claudin-5. Although the mRNA levels of ZO-1 were not altered, the protein expression was downregulated in Add-KO cells (Fig. 2A,B), on the other hand, the mRNA and protein levels of occludin were significantly elevated (Fig. 2A,B). Given the drastic drop in claudin-5 expression caused by Add loss, we also analyzed the protein expression of its direct transcriptional regulator, FoxO1^32,33^, and found that its amount was significantly higher in Add-KO cells (Fig. 2C). Together, our results indicate that Add is required for normal endothelial barrier function through proper membrane localization and expression levels of TJ and AJ key components.

### The absence of adducin does not affect the interactions between AJ components in the VE-cadherin-based complex

**Figure 3:**
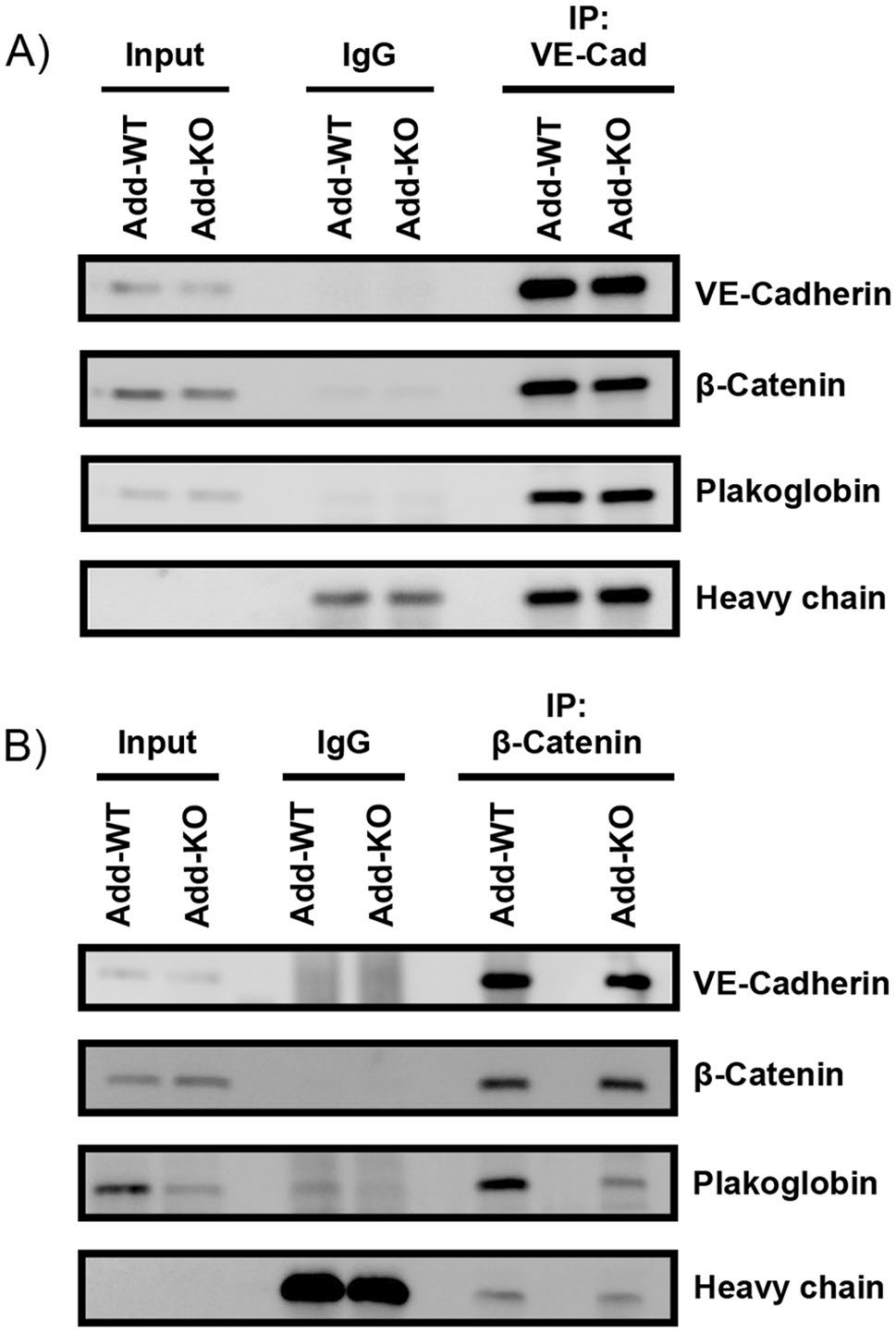
Analysis of the VE-Cadherin based complex by immunoprecipitation. Representative Western blots after (**A**) VE-Cadherin or (**B**) β-catenin pulldowns. Antibodies against VE-cadherin, ß-catenin and plakoglobin were used to asses co-immunoprecipitation partners after Add knock-out. Cell lysates were incubated with only beads and a species-specific anti-goat or anti-mouse IgG as negative controls, respectively. As input, total cell lysates before immunoprecipitation were loaded in the first and second lanes (N = 3 per group).

Since the absence of Add impaired the distribution and protein expression of junctional components, we speculated that Add could be required for their interaction within the VE-Cadherin-based complex. To prove this, immunoprecipitations of VE-Cadherin and β-catenin were performed. Similar to others, we found that both proteins interact with each other as well as with plakoglobin^34^. The interactions between VE-Cadherin/β-catenin/plakoglobin were not affected in Add-KO endothelium (Fig. 3A–B). The data indicate that Add is relevant for the localization of these proteins towards the junctions but does not modulate the interaction between them.

### Adducin is necessary for efficient re-distribution of junctional molecules at cell contacts

**Figure 4:**
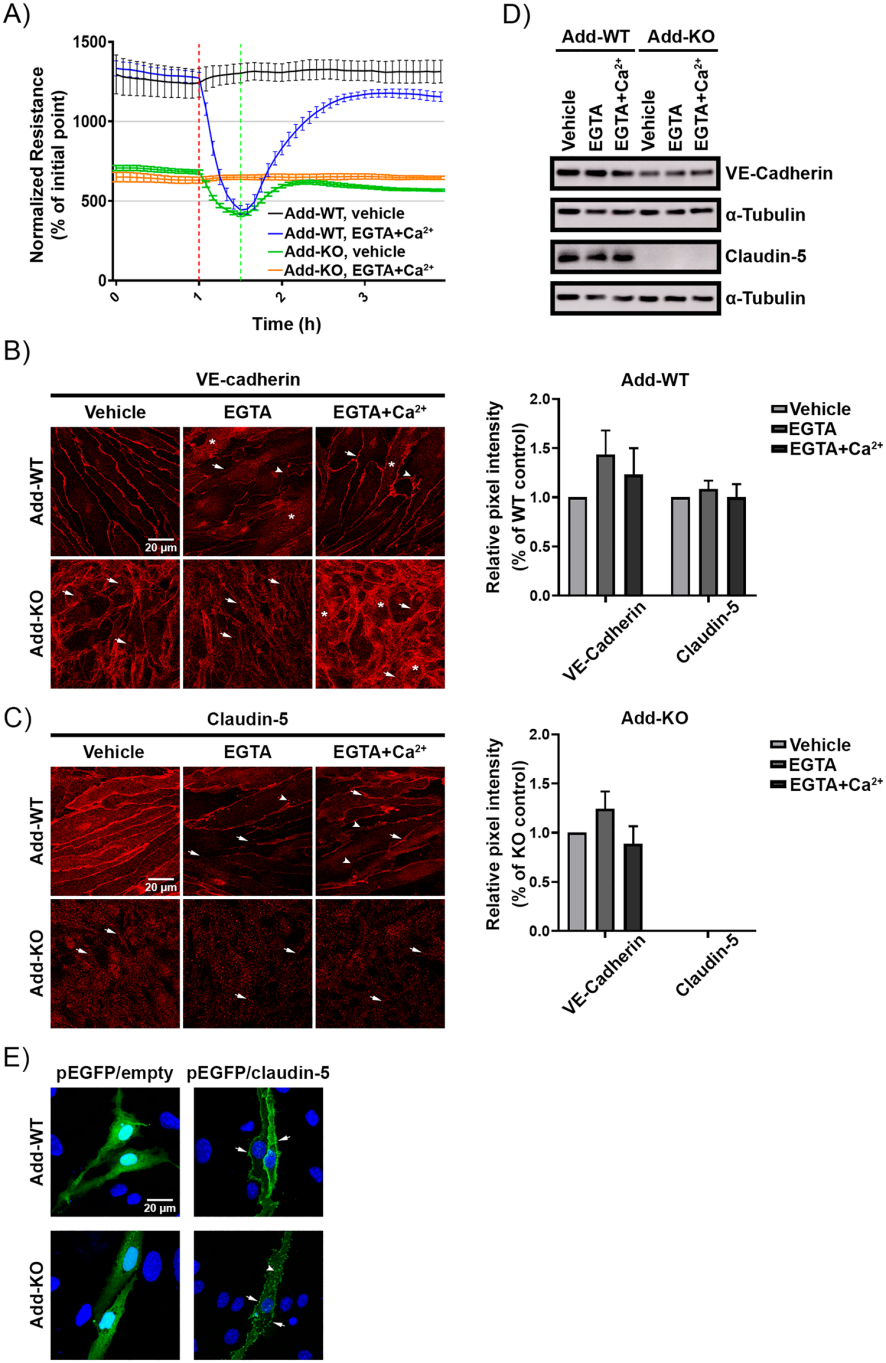
Role of Adducin in Ca^2+^-mediated recovery of endothelial barrier integrity. (**A**) Time course of TER changes in cell monolayers derived from WT and Add-KO cells subjected to Ca^2+^ switch. Addition of EGTA initiate disorganization of Ca^2+^-dependent junctions (red-dotted line). Reconstitution of Ca^2+^ engages recovery of Ca^2+^-dependent junctions (green-dotted line) (N = 4). (**B**) Immunostaining for VE-cadherin and **C)** claudin-5 of WT and Add-KO cell monolayers subjected to Ca^2+^ switch (N = 4). (**D**) Western blot analysis of VE-cadherin and claudin-5 protein expression after Ca^2+^ switch (N = 6). The bar diagrams represent the densitometric analysis of the respective blots. **E)** Representative confocal images of WT and Add-KO cells transiently transfected with either empty or pEGFP-claudin-5 vectors. The nuclei were stained using DAPI (N = 3).

To determine if Add is required during AJ or TJ assembly, confluent monolayers were subjected to calcium switch assay. First, we determined the barrier function after chelation of calcium. Addition of EGTA resulted in an immediate drop of TER in both WT and Add-KO cells, however the reduction of resistance observed in WT was more drastic, since the initial mean TER value was much higher than that of Add-KO cells. Importantly, the minimum resistance detected was similar between both cell lines. Right after calcium addition the resistance raised in both cell types; although WT cells reacted faster and the increase was stronger. The TER values achieved after calcium application remained stable until the experiment end (Fig. 4A). These effects were further analyzed by immunostainings of VE-cadherin and claudin-5. In WT cells, treatment with EGTA drastically affected the membrane localization of VE-cadherin, inducing a thin membrane pattern, gap formation and high intracellular signal (Fig. 4B, arrow, arrowhead and asterisk, respectively). In Add-KO cells, the narrow and fragmented VE-cadherin membrane staining observed under control conditions shifted to punctuated junctional distribution (Fig. 4B, arrows). In WT cells, addition of calcium induced recovery of VE-cadherin junctional signal, although some intracellular staining and zipper-like pattern remained visible (Fig. 4B, asterisk and arrowhead, respectively). In cells without Add, calcium administration caused a strong intracellular accumulation of VE-cadherin signal and junctional gaps were still visible (Fig. 4B, asterisks and arrows, respectively). Similarly to VE-cadherin, the claudin-5 staining after EGTA treatment was thinner and formation of junctional gaps and vesicle-like dots located close to the membrane were evident (Fig. 4C, arrows and arrowhead, respectively). Cells lacking Add showed little to none membrane distribution of claudin-5 under control conditions that became less apparent after treatment with EGTA (Fig. 4C, arrows). Introduction of calcium engaged recovery of claudin-5 signal at the membrane of WT cells, demonstrating a more regular staining along the monolayer, despite the presence of some gaps (Fig. 4C, arrows and arrowheads, respectively). On the other hand, no obvious differences could be detected in cells without Add, where the claudin-5 signal was similar to the one present in vehicle- and EGTA-treated monolayers (Fig. 4C). In addition, the overall protein levels of VE-cadherin and claudin-5 were investigated and no significant changes were detected for both proteins in any of the conditions tested either in WT or Add-KO (Fig. 4D).

Since the visualization of claudin-5 in cells lacking Add was challenging, transient transfection with EGFP-tagged claudin-5 was performed in sub-confluent WT and Add-KO cells. An empty EGFP vector was used as a control. WT and Add-KO cells transfected with the control plasmid showed a strong cytoplasmic and nuclear EGFP signal (Fig. 4E). On the other hand, WT cells transfected with the pEGFP-claudin-5 construct demonstrated a clear continuous membrane localization of the fusion protein (Fig. 4E, arrows). In contrast, cells lacking Add showed dotted membrane distribution of claudin-5 and abundant vesicle-like intracellular signal accumulation (Fig. 4E, arrows and arrowhead, respectively). Our data suggests that Add is necessary for proper membrane translocation of VE-cadherin and claudin-5 in newly forming cell junctions.

### cAMP-mediated barrier strengthening requires the presence of adducin but does not modulate the intracellular cAMP concentration

**Figure 5:**
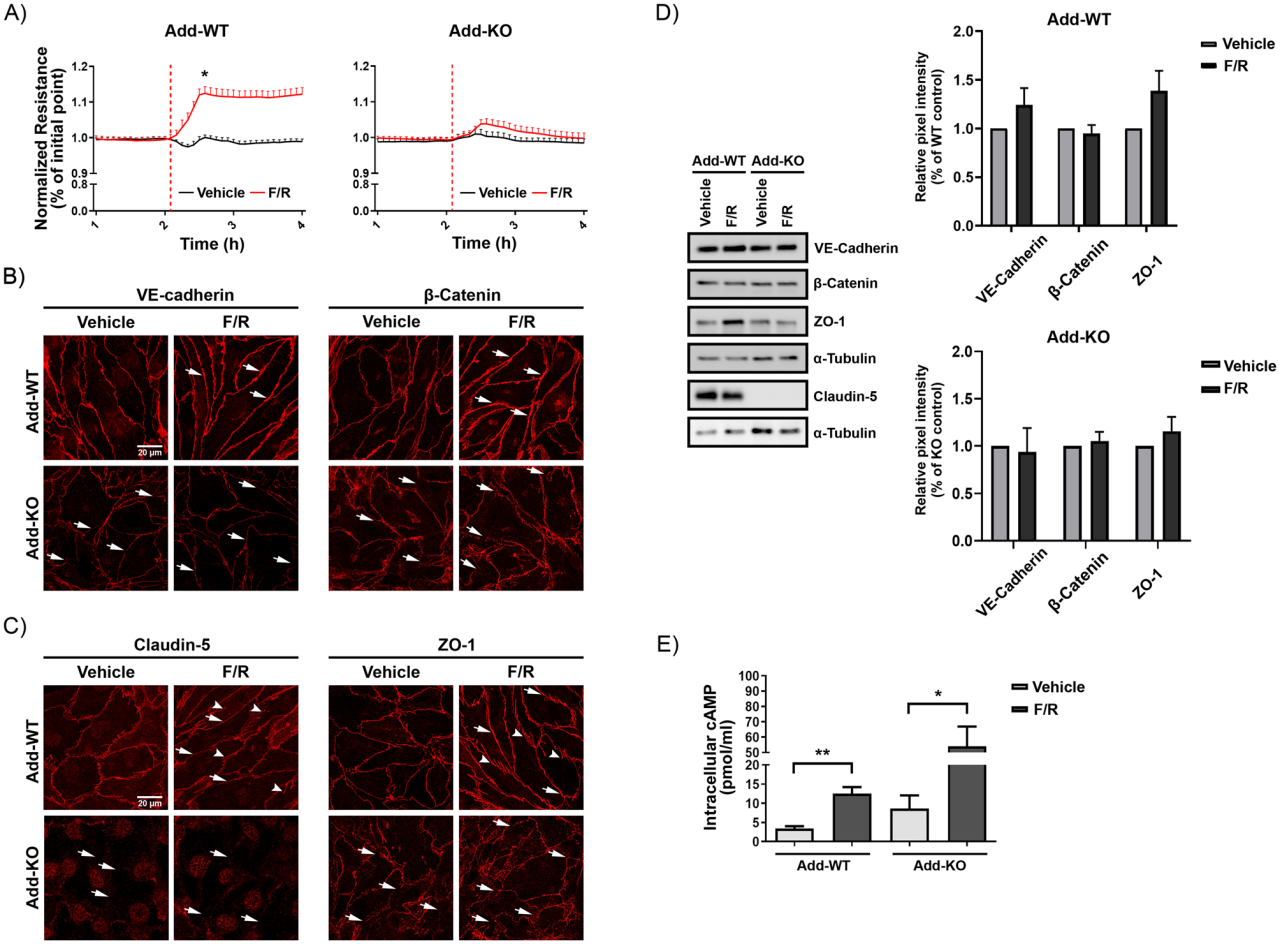
Effect of Adducin on cAMP-mediated endothelial barrier response. (**A**) Confluent WT and Add-KO cell monolayers were treated with Vehicle or F/R. The TER was measured up to 3 h after the treatment. The red-dashed line indicates the time of application. Data are normalized to the initial point of each corresponding group. “*” denotes significant difference between Vehicle and F/R in WT cells (N = 3 per group). Immunostainings for (**B**) VE-cadherin, β-catenin and (**C**) claudin-5, ZO-1 in WT and Add-KO cells treated for 1 h with either Vehicle or F/R. In WT cells arrows notice areas on the membrane where thicker signal is observed, arrowheads highlight finger-like projections. In KO cells arrows indicate fragmented junctional staining (N = 3–4 per group). (**D**) Representative Western blots for key AJ and TJ components from confluent WT and Add-KO cell monolayers exposed to Vehicle or F/R for 1 h. The graphs represent the relative pixel intensity of the bands, normalized to the WT, vehicle controls (N = 4 per group). (**E**) The graph shows intracellular cAMP concentration in pmol/ml from confluent endothelial cells monolayers analyzed by cAMP-specific ELISA (N = 8 per group). Data are represented as mean ± SEM; *p < 0.05; **p < 0.01.

The relevance of cAMP for endothelial barrier function is well known^27,35–37^, however, whether Add is associated with cAMP-mediated barrier stabilization has not been investigated. Based on our data thus far, we speculated that in contrast to WT, Add-KO endothelium may have disturbed intracellular cAMP signaling. Therefore, we used forskolin (adenylyl cyclase activator) in combination with rolipram (phosphodiesterase 4 inhibitor) to stimulate intracellular cAMP increase^27^. As expected, WT endothelial cells reacted with stronger barrier resistance shortly after forskolin/rolipram (F/R) application (Fig. 5A). In contrast, Add-KO cells did not react to this stimulus (Fig. 5A), indicating that Add is required for cAMP-mediated endothelial barrier enhancement.

Next, the distribution of TJs and AJs molecules following Vehicle or F/R application was investigated. We found that Add is potentially important for cAMP-mediated junctional re-organization, as in WT cells treated with F/R, the VE-cadherin and β-catenin signals appeared more prominent in some junctional areas (Fig. 5B, arrows). On the other hand, the localization pattern of those AJ molecules remained unchanged in cells without Add, where a fragmented membrane signal persisted (Fig. 5B, arrows). The response to the intracellular cAMP increase in WT cells was also favorable for TJ components since the claudin-5 staining at cell junctions was more regular (Fig. 5C, arrows). In contrast, cells lacking Add showed no claudin-5 reorganization after F/R, exhibiting areas with missing claudin-5 signal and drastically fragmented staining for the tight junctional protein, similarly to the vehicle-treated cells (Fig. 5C, arrows). Interestingly, in WT monolayers induction of cAMP changed the pattern of ZO-1 from a thin and semi-discontinuous junctional distribution to partially thicker staining in combination with finger-like extensions (Fig. 5C, arrows and arrowheads, respectively). However, the junctional distribution of ZO-1 in Add-KO cells appeared irregular and fragmented and no important effect was observed following F/R application (Fig. 5C, arrows). The protein level of the above-mentioned molecules after induction of intracellular cAMP was also investigated and no significant alterations were detected in WT cells (Fig. 5D). The outcome was similar for Add-KO cells (Fig. 5D). We hypothesized that the lack of cAMP-mediated barrier strengthening and junctional reorganization seen in Add-KO cells was associated with altered cAMP concentration; therefore, a cAMP ELISA assay was performed. Surprisingly, under control conditions, the difference in the cAMP concentration between WT and Add-KO was not prominent. Nevertheless, in both cell lines, the response to F/R treatment significantly increased the intracellular cAMP (Fig. 5E). The data suggest that Add does not control the release of cAMP.

In addition, it is well known that cAMP-mediated signaling activates Epac1 resulting in barrier enhancement^38^. For this reason, we tested whether Add affects the Epac1-dependent barrier augmentation. Thus, WT and Add-KO monolayers were stimulated with the specific Epac1 activator “007”. Similar to F/R treatment, stimulation with 007 induced a modest but significant increase in the resistance for WT cells only (Supplementary Fig. 1A). Additionally, application of 007 had no important effect on claudin-5 distribution in both cell lines (Supplementary Fig. 1B). This suggest that Add is required for proper Epac1 signaling and that Epac1 activation alone does not seem to play a critical role in junctional redistribution of claudin-5.

### Lack of adducin leads to increased RhoA activity and the presence of Adducin appears to be necessary to induce efficient cAMP-mediated Rac1 activation

**Figure 6:**
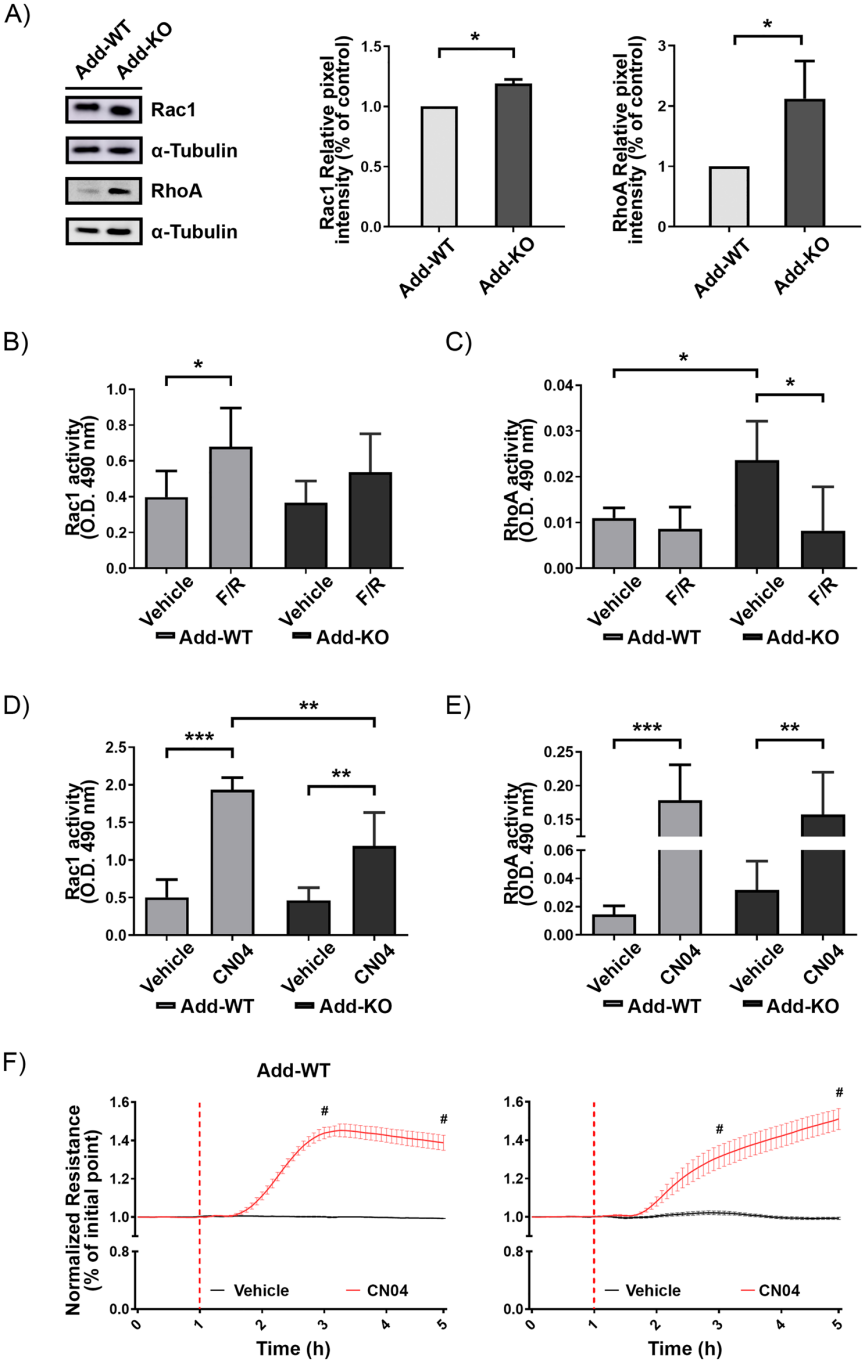
Protein expression, activation state of Rac1 and RhoA small GTPases and GTPase activity effects on TER. (**A**) Representative Western blot images for Rac1 and RhoA under basal conditions and respective pixel intensity quantifications presented as bar graphs. The data are normalized to the corresponding WT control (N = 4 per group). G-LISA small GTPases activation analysis of (**B**) Rac1 and (**C**) RhoA from confluent WT and Add-KO cells treated with Vehicle or F/R for 1 h. Activation status of (**D**) Rac1 and (**E**) RhoA from cells stimulated with Vehicle or CN04 for 2 h. Bar graphs show raw optical density (O.D.) measured with a wavelength of 490 nm (N = 4–7 per group). (**F**) TER measurements from confluent WT and Add-KO cell monolayers. Red-dashed line indicates the time of treatments. Data are normalized to the initial point of each corresponding group. “#” denotes statistically significant difference between Vehicle and CN04 treated groups (N = 3 per group). Data are represented as mean ± SEM; *p < 0.05; **p < 0.01; ***p < 0.001, # *p* < 0.0001.

Since regulation of the endothelial barrier is known to be directly affected by the activity of small Rho GTPases such as Rac1 and RhoA^39–41^, we investigated if Add would affect the protein regulation or activation state of these molecular switches. Interestingly, Add-KO showed significantly higher expression of Rac1 and RhoA proteins (Fig. 6A). The activation of Rac1 and RhoA was investigated by G-LISA. Our data showed that the activity of Rac1 was similar in both vehicle-treated cell lines. Furthermore, only WT cells displayed a significant activation of Rac1 after F/R application (Fig. 6B), suggesting that Add may be necessary for cAMP-mediated Rac1 activation. On the other hand, Add-KO cells had a strong increase in RhoA activity under control conditions when compared to WT cells. However, this elevation was blunted upon F/R treatment, indicating that cAMP-mediated signaling is sufficient to reduce RhoA activity, possibly in a Rac1-independent manner (Fig. 6C).

### The direct activation of Rac1 and RhoA is not affected by Adducin and leads to enhanced barrier function

Considering our previous results, we investigated if the lack of Add affects the stability of Rac1 and RhoA molecules by employing the synthetic Rac1/RhoA/Cdc42 activator I (CN04), which directly triggers Rho GTPase by deamidating glutamine-63 of Rho and glutamine-61 of Rac1 and Cdc42^42,43^. We discovered that Rac1 activation could be induced by CN04 in both cell lines, although this effect was significantly lower in Add-KO cells compared to WT controls (Fig. 6D), further suggesting that Add is upstream of Rac1. However, Add was not required for direct activation of RhoA via the bacterial toxin, as the activation induced by CN04 was comparable between both cell lines (Fig. 6E). Lastly, we investigated if the simultaneous triggering of Rac1 and RhoA would have any impact on the cell monolayer integrity. Surprisingly, the application of CN04 strongly increased the TER regardless of the presence or absence of Add (Fig. 6F), showing that once these GTPases are activated, Add is not required to exert their function. In addition, indirect activation of RhoA via calpeptin (CN01) resulted in diminished barrier function in both WT and Add-KO cells, suggesting that the CN04-mediated effect is mostly related to Rac1 but not RhoA activity (Supplementary Fig. 2). Treatment with CN01 did not affect the activation state of Rac1 (data not shown).

## Discussion

Several studies have acknowledged Add as critical structural component in erythrocytes and neurons necessary to stabilize actin/spectrin networks and new synapse formation, respectively^44–49^. Considering these studies and given its cortical localization and interaction with actin networks, Add may have potential critical functions in cell–cell junctional stabilization. In this regard, it has been shown that Add regulates the remodeling of epithelial cells apical junctions^50^, desmosomal cohesion^24^ and the interaction between desmoglein 3 and epithelial cadherin (E-Cadherin) in keratinocytes^25^. Moreover, our group previously investigated whether Add was relevant in the endothelium and found that these cells express α- and γ-Adducin. Importantly, downregulation of α-Add resulted in reduced TER associated with disrupted VE-Cadherin membranal localization and hindered barrier recovery after Ca^2+^-depletion^26^. In the current study, we further investigated the mechanism behind Add-mediated endothelial barrier disruption by utilizing MyEnds isolated from WT and Add-KO mice. We confirmed that MyEnds lacking Add had significantly reduced TER and fragmented VE-Cadherin junctional staining. The results show that besides the changes in the localization of VE-Cadherin, its protein levels were considerably reduced. Moreover, the expression and localization pattern of other key AJ and TJ components such as β-catenin, plakoglobin, claudin-5, occludin and ZO-1 were significantly altered (Figs. 1 and 2). Of great importance, however, is the downregulation of VE-Cadherin in cells without Add, as the formation of VE-Cadherin-mediated contacts can upregulate the expression of claudin-5 by blocking FoxO1 function^51^. Our results showed that the lack of Add not only led to an almost complete downregulation of claudin-5 and its mRNA levels, but also increased the protein expression of occludin, possibly as part of a compensatory mechanism to preserve barrier properties (Fig. 2). The negative regulation of claudin-5 could be explained by the novel finding reported in this study, where the absence of Add induces high expression of FoxO1, a very-well known claudin-5 transcriptional repressor^32,33,51^. In this context, it is reported that Add can interact with the RNA polymerase II and different transcriptional regulators such as the zinc finger protein 331 (ZNF331) or regulatory factor X1 (RFX1)^52,53^. Therefore, a potential direct connection between Add and FoxO1 could exist. On the other hand, this could be in part, mediated via VE-Cadherin-dependent AJ formation but the details behind these pathways remain to be elucidated in endothelial cells. The notion that Add is an important junctional regulator is supported by previous studies in human epithelial cells, where it was demonstrated that loss of Add leads to less dense cortical actin and subsequent diminished interaction of desmoglein 3 and epithelial cadherin (E-cadherin), and therefore affecting epithelial junction turnover^25,50^. We found that the absence of Add does not disturb the molecular interactions within the VE-Cadherin-based junctional complex (Fig. 3). Thus, with the current data set we can speculate that Add may serve as a bridge between these proteins and the actin cytoskeleton to stabilize the cell–cell interactions. Such idea has been previously proposed in erythrocytes, where Add serves as the link between the band 3 protein and actin to stabilize the membrane^46^. We also demonstrate that Add is required for the proper localization of VE-cadherin and claudin-5 in forming cell junctions, since calcium depletion and repletion in Add-KO cells led to intracellular accumulation of VE-cadherin, no changes in the redistribution of claudin-5 and slower resistance recovery (Fig. 4). Transfection of sub-confluent cell monolayers with pEGFP-claudin-5 construct revealed that cells without Add were unable to efficiently translocate claudin-5 at the junction. Here, vesicle-like green dots accumulated within the intracellular region were visualized (Fig. 4). Together, our data highlights Add as a key component in maintaining proper endothelial barrier integrity, modulating the expression of AJ and TJ molecules and their localization to intercellular contacts.

As previously shown, the endothelial barrier integrity is enhanced by activation of cAMP-Epac1/Rap1 and/or –PKA-dependent pathways^27,35,36,38,54–56^. Moreover, Add function can be controlled in part by protein kinase A (PKA)-derived phosphorylation events^57,58^, suggesting a potential link between cAMP-mediated signaling and Add. For this reason, we induced an intracellular cAMP increase in WT and Add-KO MyEnds using F/R. We found that the cells without Add failed to increase barrier resistance as the WT cells did (Fig. 5). A similar outcome was observed after specific activation of Epac1-mediated signaling (Supplementary Fig. 1). Moreover, cells lacking Add were incapable of properly translocating junctional molecules to the membrane after the F/R treatment, explaining the lack of reaction observed in TER analyses. However, no significant differences were observed at the protein level in any of the conditions tested. Interestingly, our data also show that Add plays no role in the intracellular modulation of cAMP levels per se. Therefore, we can conclude that Add is not only important for the maintenance of basal endothelial barrier function but is also necessary for the cAMP-mediated accumulation of AJs and TJs proteins at the membrane.

As mentioned above, cAMP leads to modulation of Epac1/Rap1 and PKA signaling pathways, which directly affect the activation state of the small Rho GTPases Rac1 and RhoA^37,59–61^. While the activation of the RhoA/ROCK pathway has been extensively associated with endothelial cells dysfunction^39,62–64^, Rac1 stimulation is known to enhance endothelial barrier properties^10,40^. For this reason, we also evaluated the status of Rac1 and RhoA in WT and Add-KO cells. Under control conditions, the protein level of both GTPases was upregulated in cells lacking Add. In these cells, the activity of RhoA was also significantly increased whereas the Rac1 activation was comparable between both cell lines (Fig. 6). This could also explain, partially, why the absence of Add leads to compromised barrier function. Although, it has been reported that RhoA can regulate Add function through direct phosphorylation^24^, here we provide insights that Add can in fact modulate RhoA or at the very least, cause abnormal activation when it is not present. This suggests the possibility of a regulatory loop between Add and RhoA.

As expected, we found that induction of the cAMP signaling pathway through F/R application led to increased Rac1 activity in WT cells, however, this was not the case in Add-KO cells. Thus, our data suggests that Add may be required to conduct cAMP-mediated Rac1 activation in endothelial cells. This is comparable to previous studies on the ABP VASP, which showed the requirement of VASP for proper cAMP-mediated Rac1 activation^55,56^. In addition, consistent with a previous report demonstrating that F/R treatment leads to downregulation of RhoA^65^, we observed that F/R addition completely reversed the RhoA overstimulation caused by Add lost (Fig. 6), potentially via a Rac1-independent pathway.

The above-mentioned approach was not enough to test whether the lack of Add alters the functionality of the GTPase molecules. To this end, we employed the synthetic Rac1/RhoA/Cdc42 activator I, CN04 and we could demonstrate that Add was not a determining factor affecting the activation capabilities of either Rac1 or RhoA, as both GTPases were triggered following CN04 treatment. Moreover, the simultaneous activation of these molecules by CN04 was enough to overcome the destabilization of the endothelial barrier caused by the absence of Add. These results are in line with our previous study, where the dual activation of Rac1 and RhoA could improve the barrier function of Epac1-KO endothelial cells^27^. On the other hand, indirect activation of RhoA with CN01 led to diminished barrier function (Supplementary Fig. 2), suggesting that coordinated Rac1 and RhoA activation is necessary to increase barrier resistance as observed after CN04 treatment. Altogether, these data show that cAMP-mediated endothelial barrier enhancement requires Add to efficiently activate Rac1, but does not directly affect the functionality of Rac1 or RhoA molecules. This suggests that Add is upstream of Rac1 and could be involved in its spatio-temporal regulation and thus, directly or indirectly modulate RhoA function. In contrast to this speculation, there is evidence showing that Adducin-γ, the brain isoform of Add, is regulated by Rac1 activation and RhoA inhibition following shear stress in the blood–brain barrier^66^. Hence, the precise molecular interplay taking place in this phenomenon needs to be further investigated. In summary, our study shows that Add is critical for the maintenance of endothelial cell junctions and participates in cAMP-mediated Rac1 and RhoA regulation.

## Supplementary Information

Supplementary Information.

## Data Availability

The datasets generated and/or analyzed during the current study are available upon reasonable request to the corresponding author. We do not have the means to provide a permalink for a storage database.

## Abbreviations

Add: Adducin
TJ: Tight junction
AJ: Adherens junction
TER: Transendothelial electrical resistance
cAMP: Cyclic adenosine monophosphate
IP: Immunoprecipitations
GTPase: Guanosine-triphosphatase
ELISA: Enzyme-linked immunosorbent assay
