# Supplementary material for: Lack of adducin impairs the stability of endothelial adherens and tight junctions and may be required for cAMP-Rac1-mediated endothelial barrier stabilization
Source: Sci Rep. 2022 Sep 2;12:14940. doi: 10.1038/s41598-022-18964-5 (PMC9440001; doi:10.1038/s41598-022-18964-5)
Supplement: Supplementary file 1 — Supplementary Information. [file 41598_2022_18964_MOESM1_ESM.pdf]

Lack of Adducin impairs the stability of endothelial Adherens and Tight Junctions and may be required for cAMP-Rac1-mediated endothelial barrier stabilization

*Sina Moztarzadeh ┼, Mariya Y. Radeva ┼, Sara Sepic, Katharina Schuster, Ibrahim Hamad, Jens Waschke and Alexander García-Ponce**

Chair of Vegetative Anatomy, Faculty of Medicine, Ludwig-Maximilians-University (LMU) Munich, Pettenkoferstraße 11, D-80336 Munich, Germany

┼ *these authors contributed equally*

* *corresponding author*

Tel. +49 89 2180 -72699

email. alexander.garcia@med.uni-muenchen.de

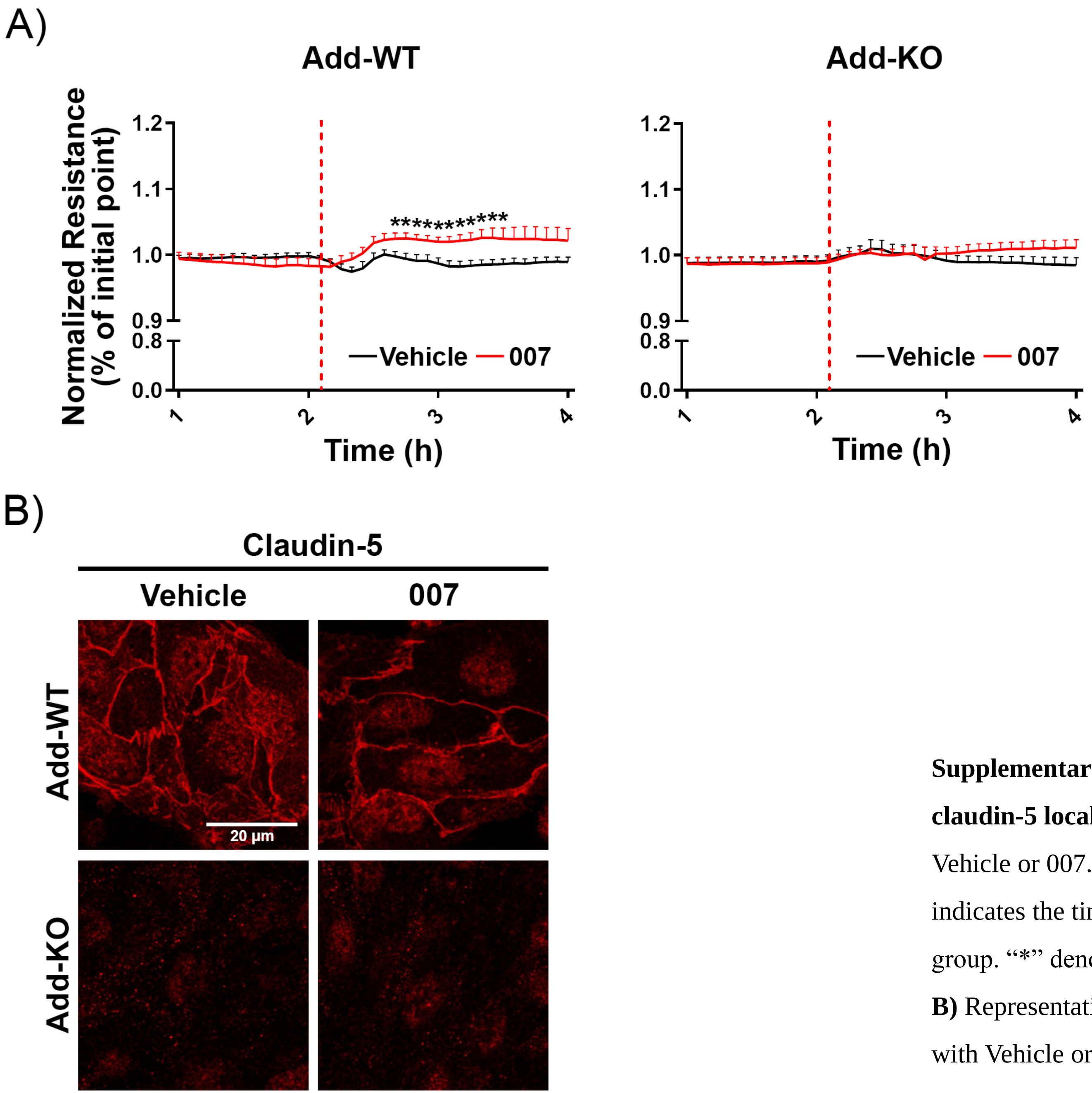

Supplementary Figure 1. Effect of specific Epac1 activation on barrier resistance and claudin-5 localization. A) Confluent WT and Add-KO cell monolayers were treated with either Vehicle or 007. The TER was measured up to 3 hours after the treatments. The red-dashed line indicates the time of application. Data are normalized to the initial point of each corresponding group. "*" denotes significant difference between Vehicle and 007 in WT cells (N=3 per group). **B)** Representative claudin-5 immunostainings in WT and Add-KO confluent monolayers treated with Vehicle or 007 (N=3).

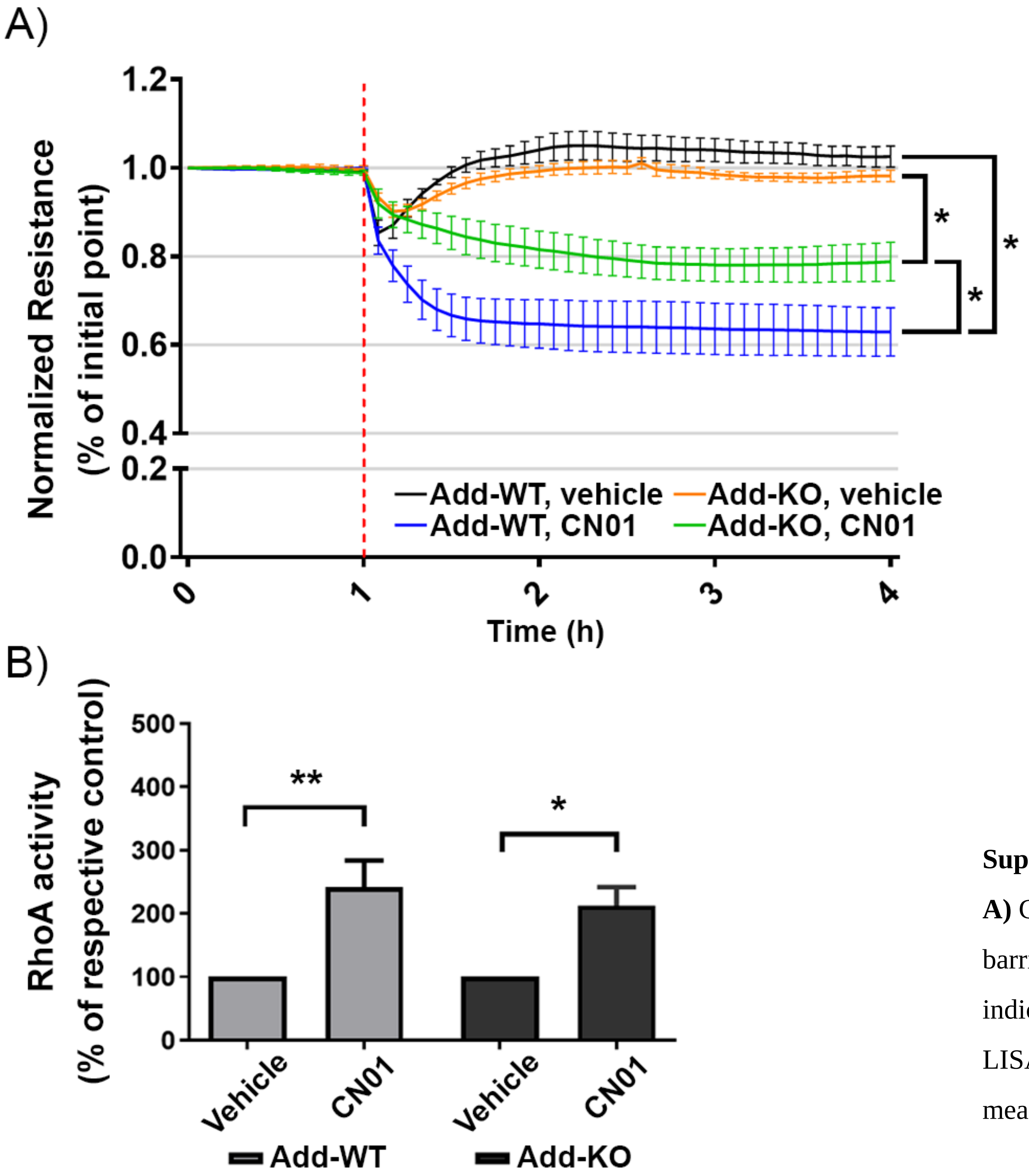

Supplementary Figure 2. Consequence of CN01 treatment on endothelial barrier function. **A)** Confluent WT and Add-KO cell monolayers were treated with either Vehicle or CN01. The barrier function was measured 1 hour before and 3 hours after treatment. The red-dotted line indicates treatment addition; *p<0.0001. **B)** Activity of the small GTPase RhoA analyzed by G-LISA. Bar graphs show the activity normalized to the respective control. Data are presented as mean ± SEM; *p<0.05; **p<0.01.

Original PCR gel images

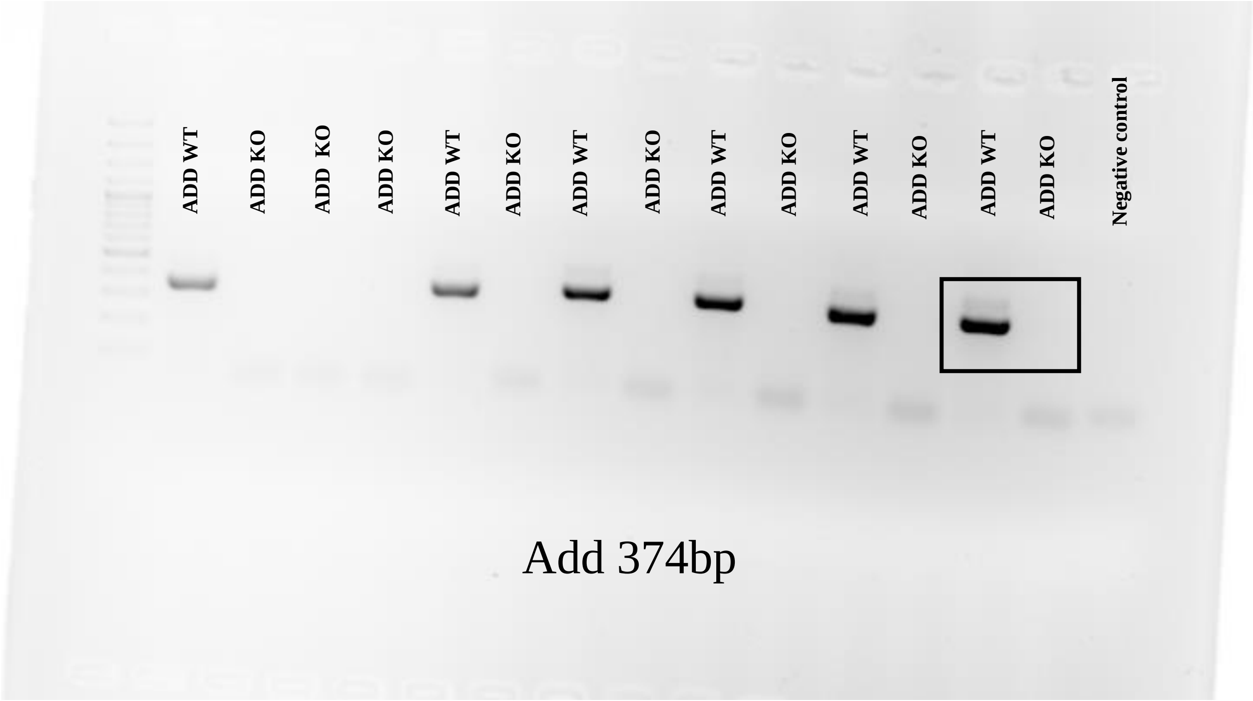
ADD WT
ADD KO
ADD KO
ADD KO
ADD WT
ADD KO
ADD WT
ADD KO
ADD WT
ADD KO
ADD WT
ADD KO
ADD WT
ADD KO
Negative control
Add 374bp

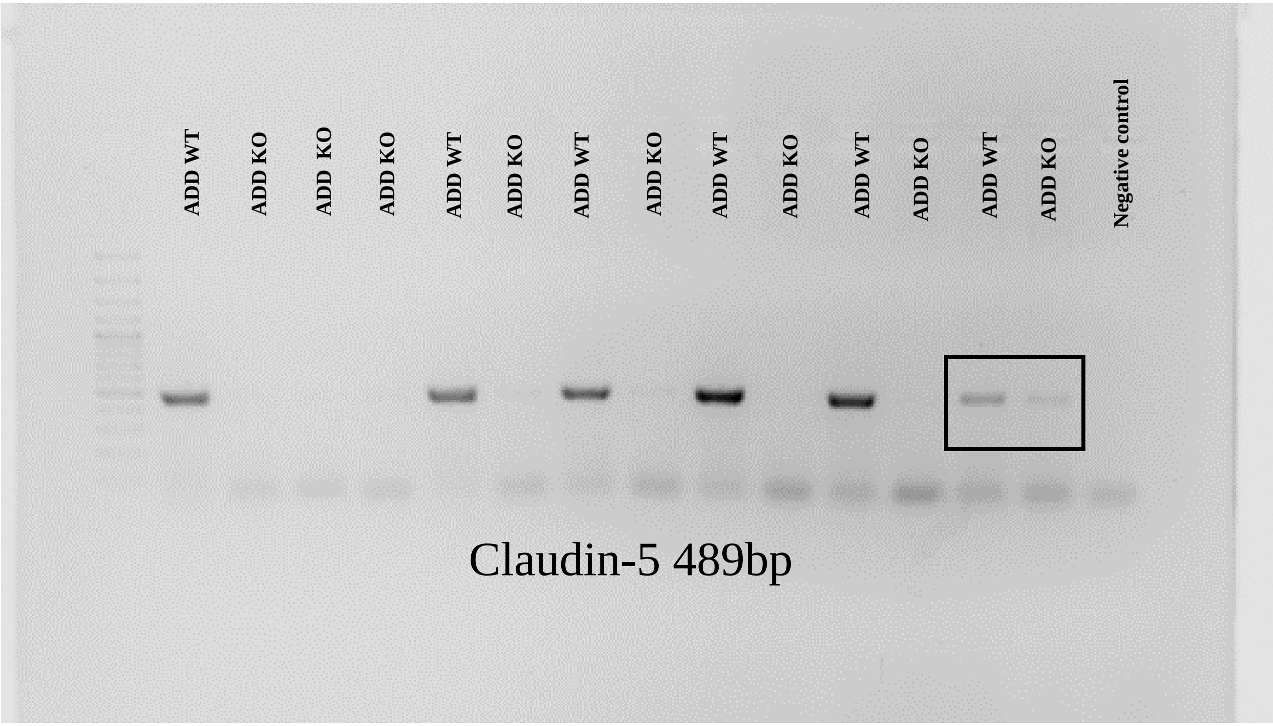
ADD WT
ADD KO
ADD KO
ADD KO
ADD WT
ADD KO
ADD WT
ADD KO
ADD WT
ADD KO
ADD WT
ADD KO
ADD WT
ADD KO
Negative control
Claudin-5 489bp

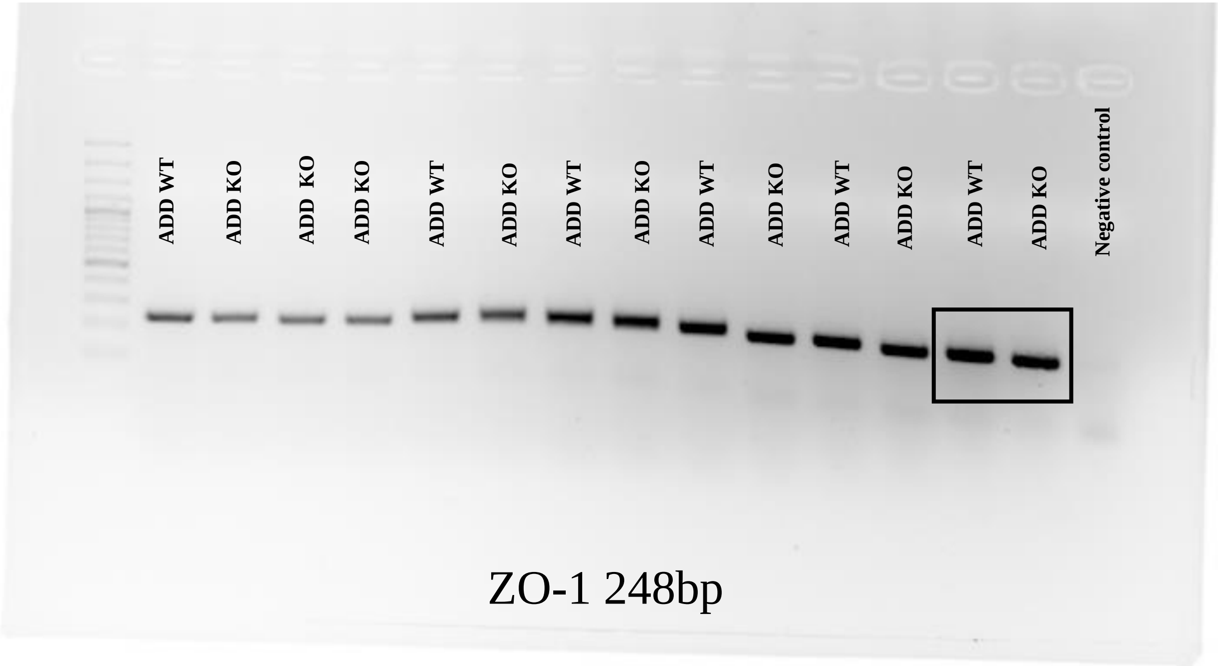
ADD WT
ADD KO
ADD KO
ADD KO
ADD WT
ADD KO
ADD WT
ADD KO
ADD WT
ADD KO
ADD WT
ADD KO
ADD WT
ADD KO
Negative control
ZO-1 248bp

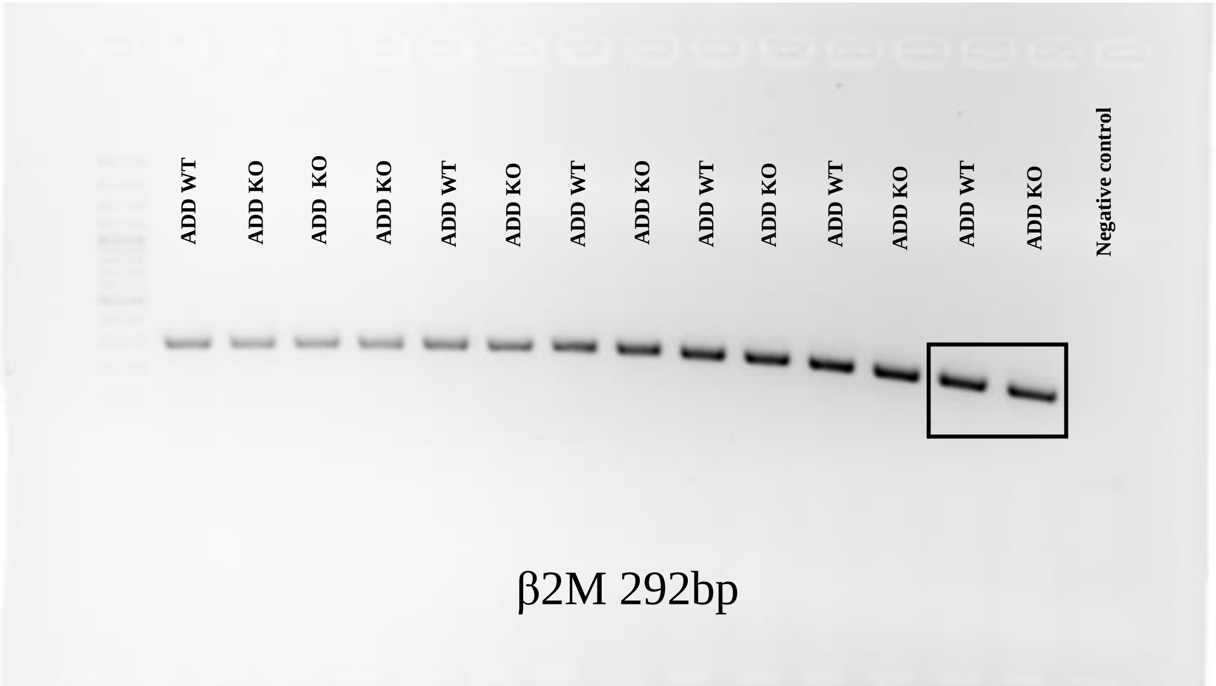
ADD WT
ADD KO
ADD KO
ADD KO
ADD WT
ADD KO
ADD WT
ADD KO
ADD WT
ADD KO
ADD WT
ADD KO
ADD WT
ADD KO
Negative control
β2M 292bp

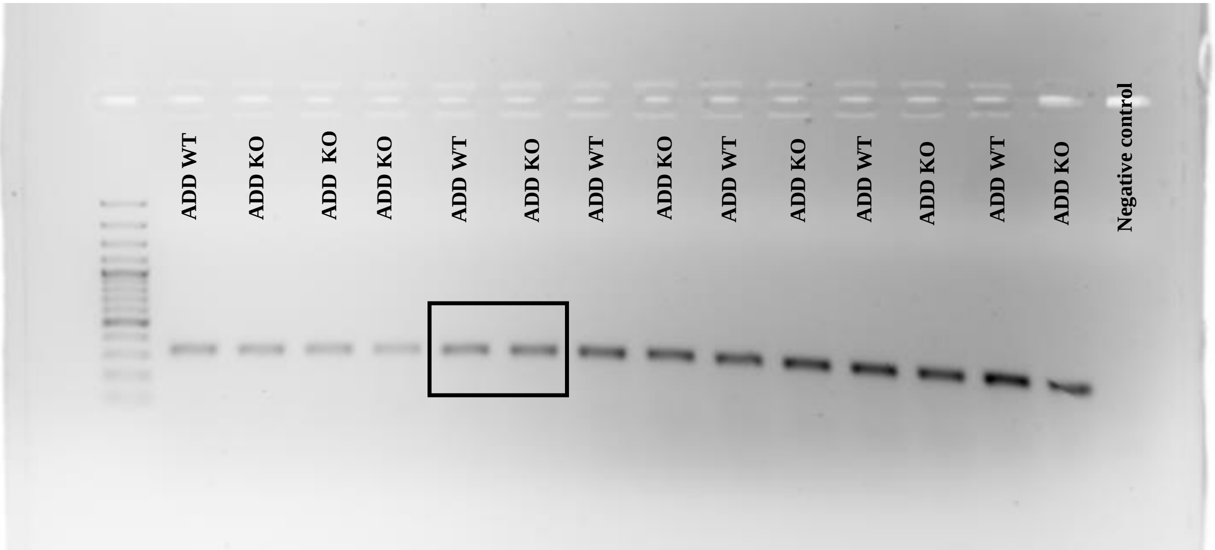

VE-cadherin 323bp

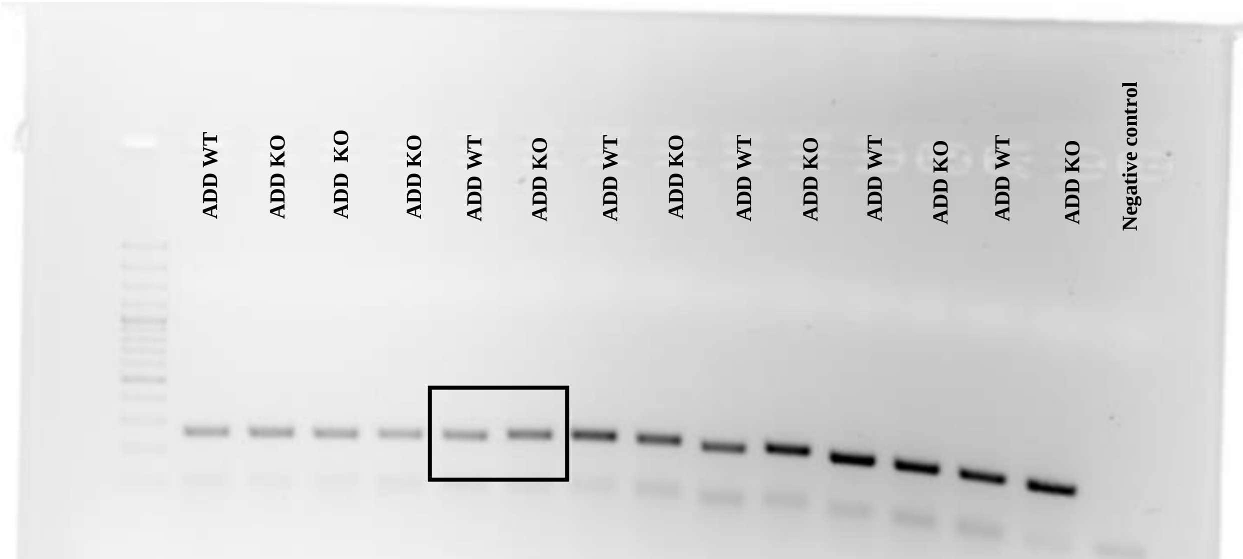

Occludin 248bp

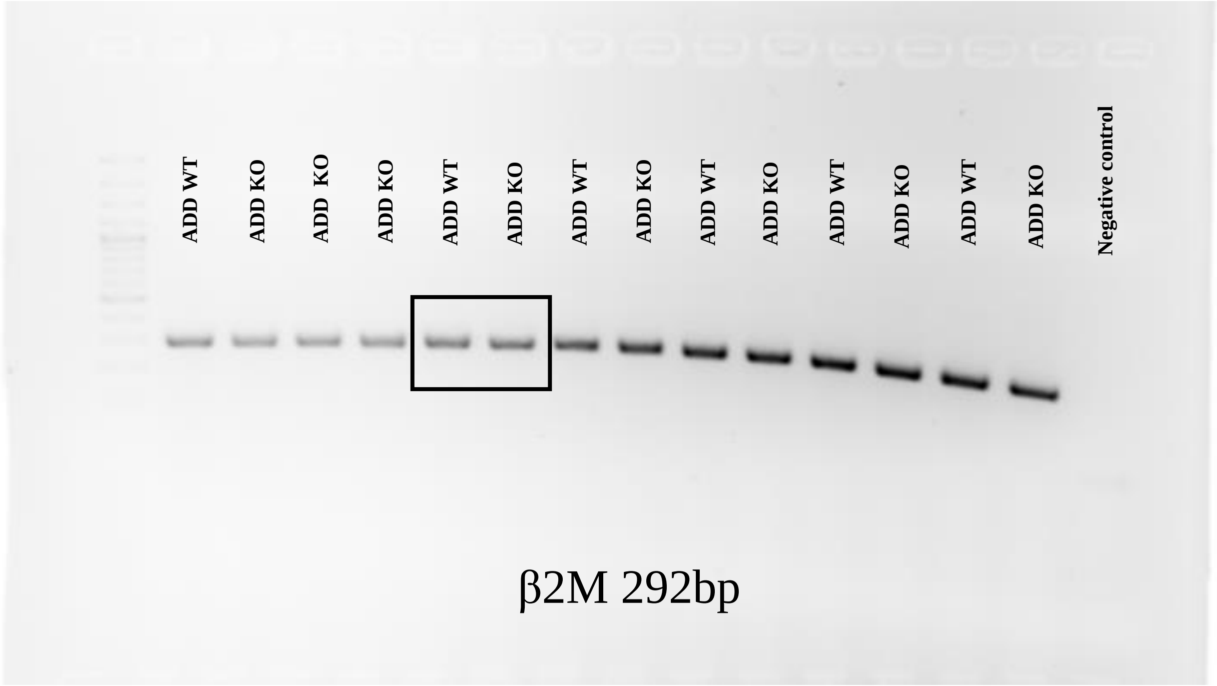
ADD WT
ADD KO
ADD KO
ADD KO
ADD WT
ADD KO
ADD WT
ADD KO
ADD WT
ADD KO
ADD WT
ADD KO
ADD WT
ADD KO
Negative control
β2M 292bp

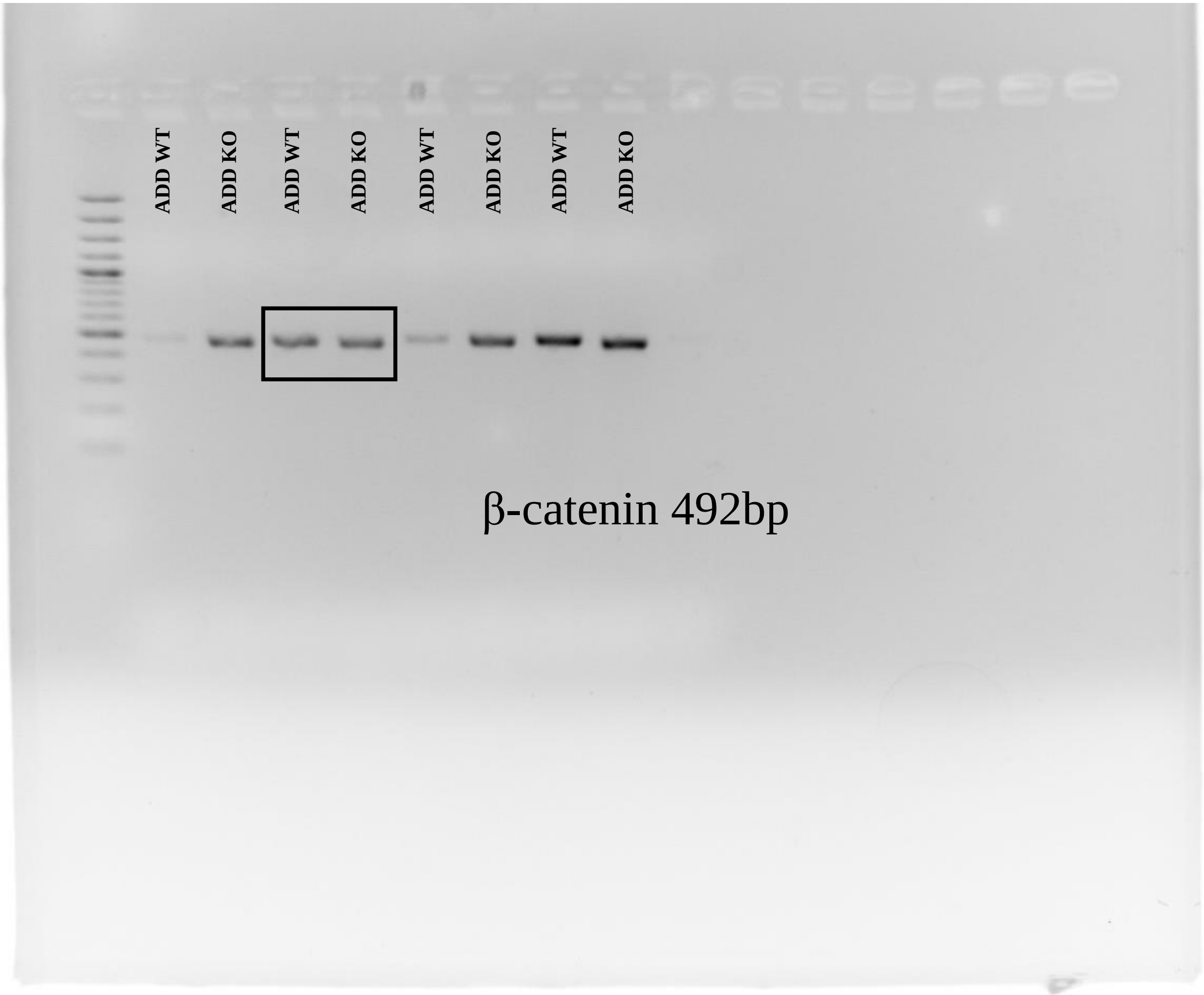
ADD WT
ADD KO
ADD WT
ADD KO
ADD WT
ADD KO
ADD WT
ADD KO
β-catenin 492bp

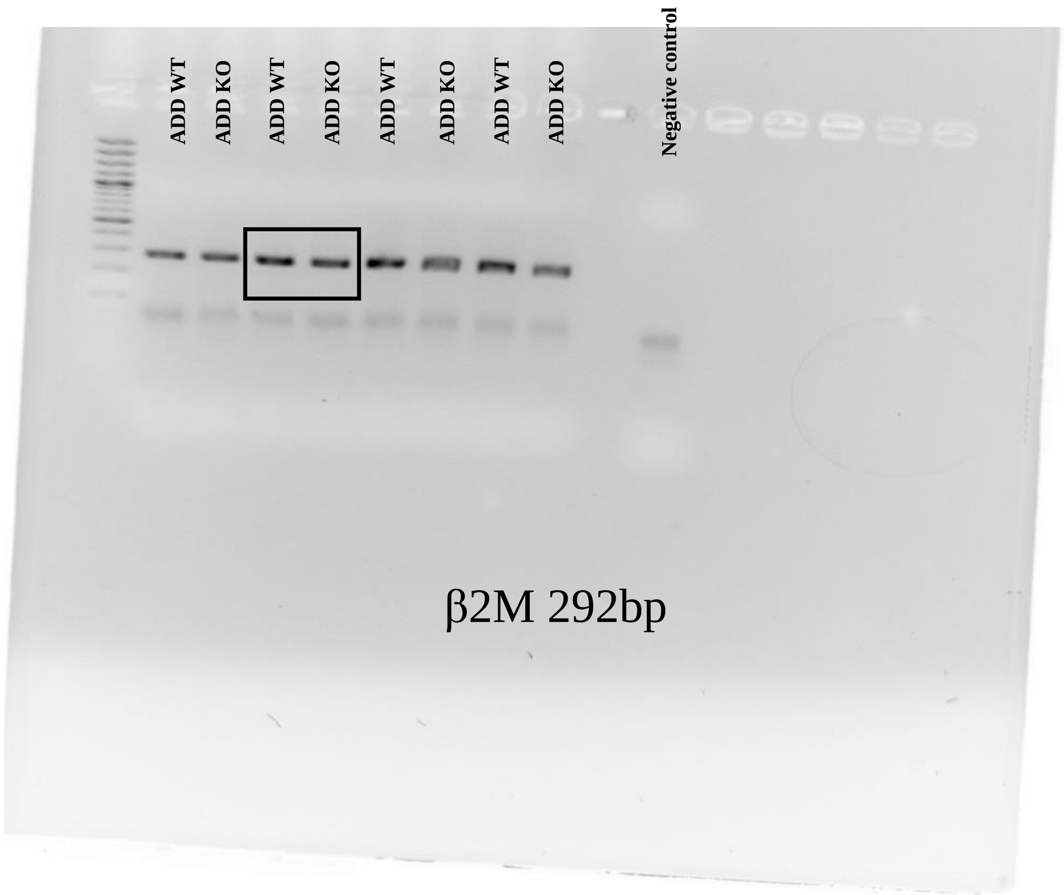
ADD WT
ADD KO
ADD WT
ADD KO
ADD WT
ADD KO
ADD WT
ADD KO
Negative control
β2M 292bp

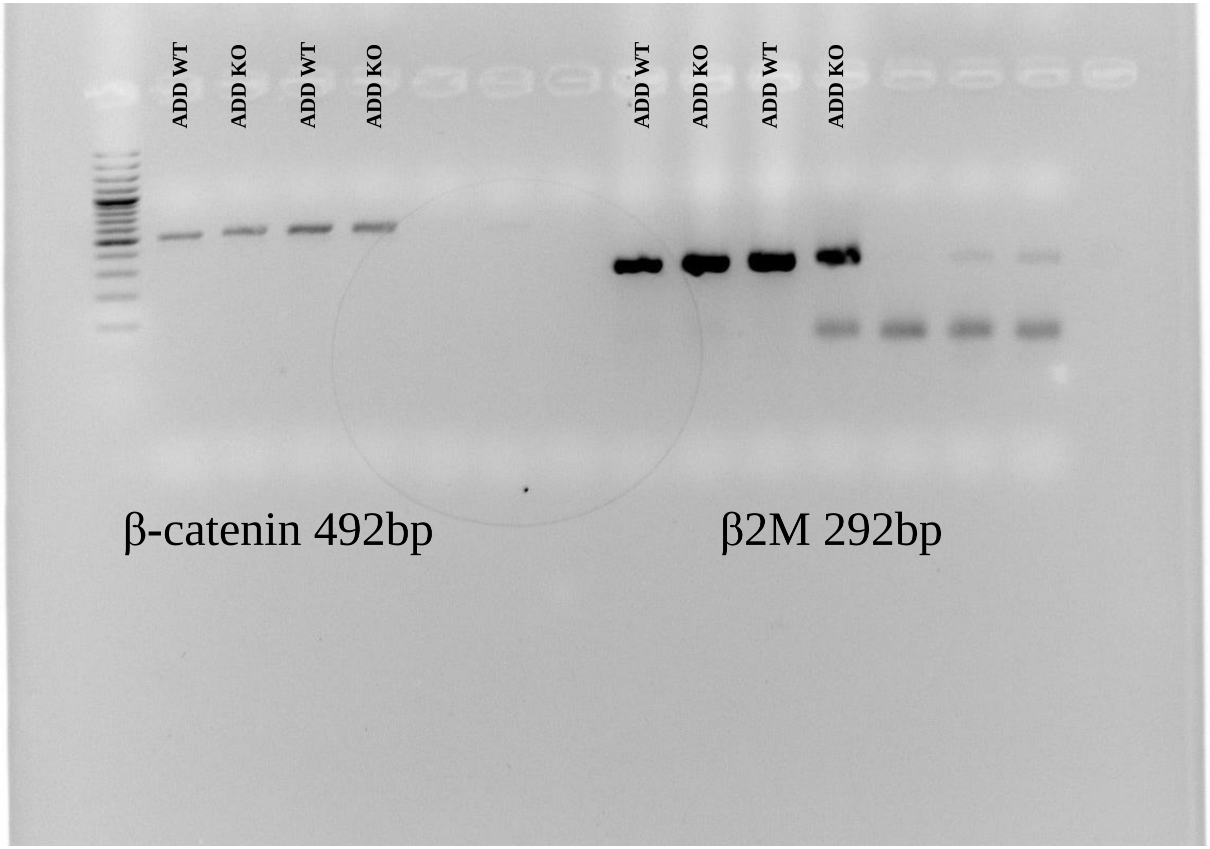
ADD WT
ADD KO
ADD WT
ADD KO
ADD WT
ADD KO
ADD WT
ADD KO
β-catenin 492bp
β2M 292bp

Original Western blot gel images for basal conditions

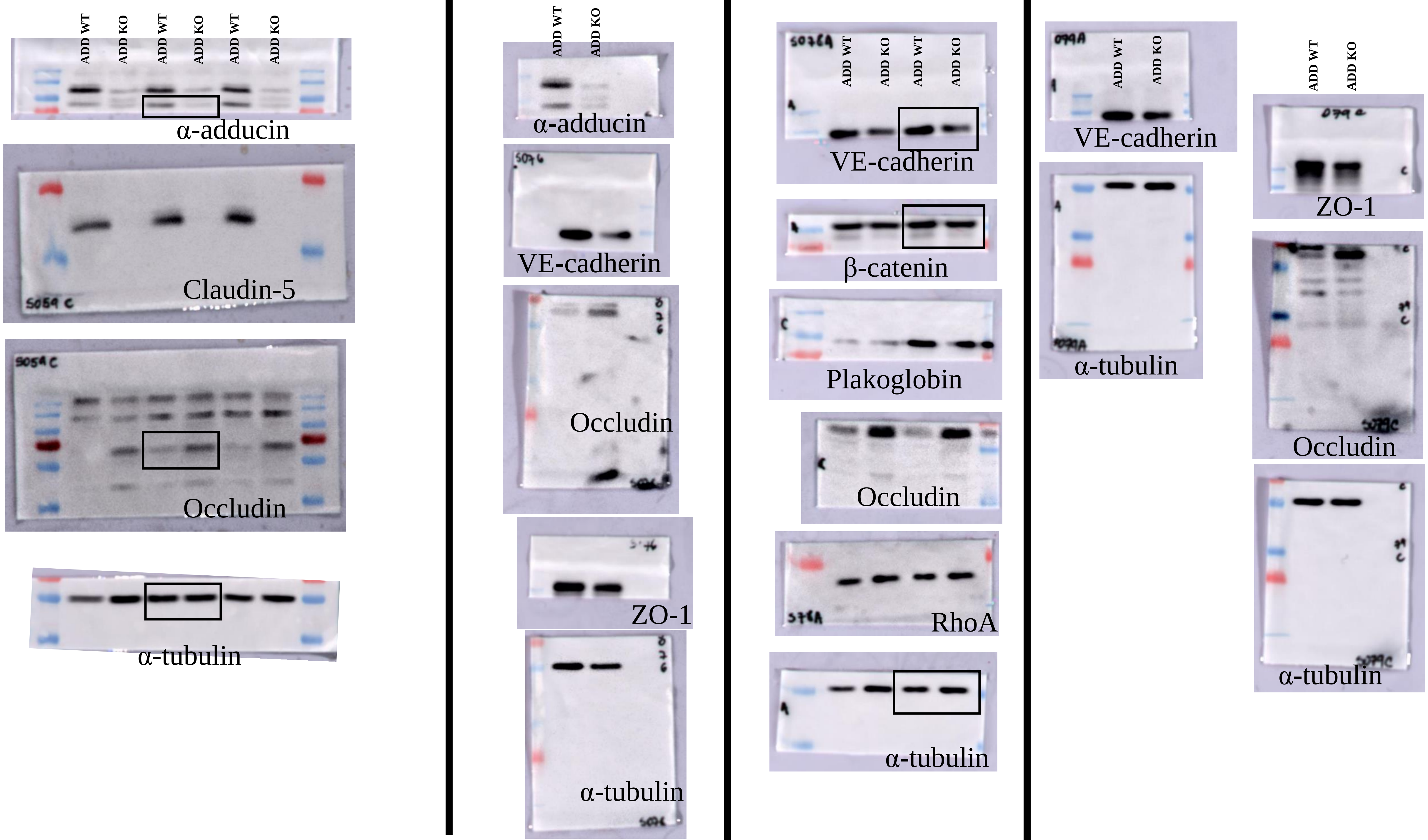
ADD WT
ADD KO
ADD WT
ADD KO
ADD WT
ADD KO
α-adducin
Claudin-5
Occludin
α-tubulin
ADD WT
ADD KO
α-adducin
VE-cadherin
Occludin
ZO-1
α-tubulin
ADD WT
ADD KO
ADD WT
ADD KO
VE-cadherin
β-catenin
Plakoglobin
Occludin
RhoA
α-tubulin
ADD WT
ADD KO
VE-cadherin
α-tubulin
ADD WT
ADD KO
ZO-1
Occludin
α-tubulin

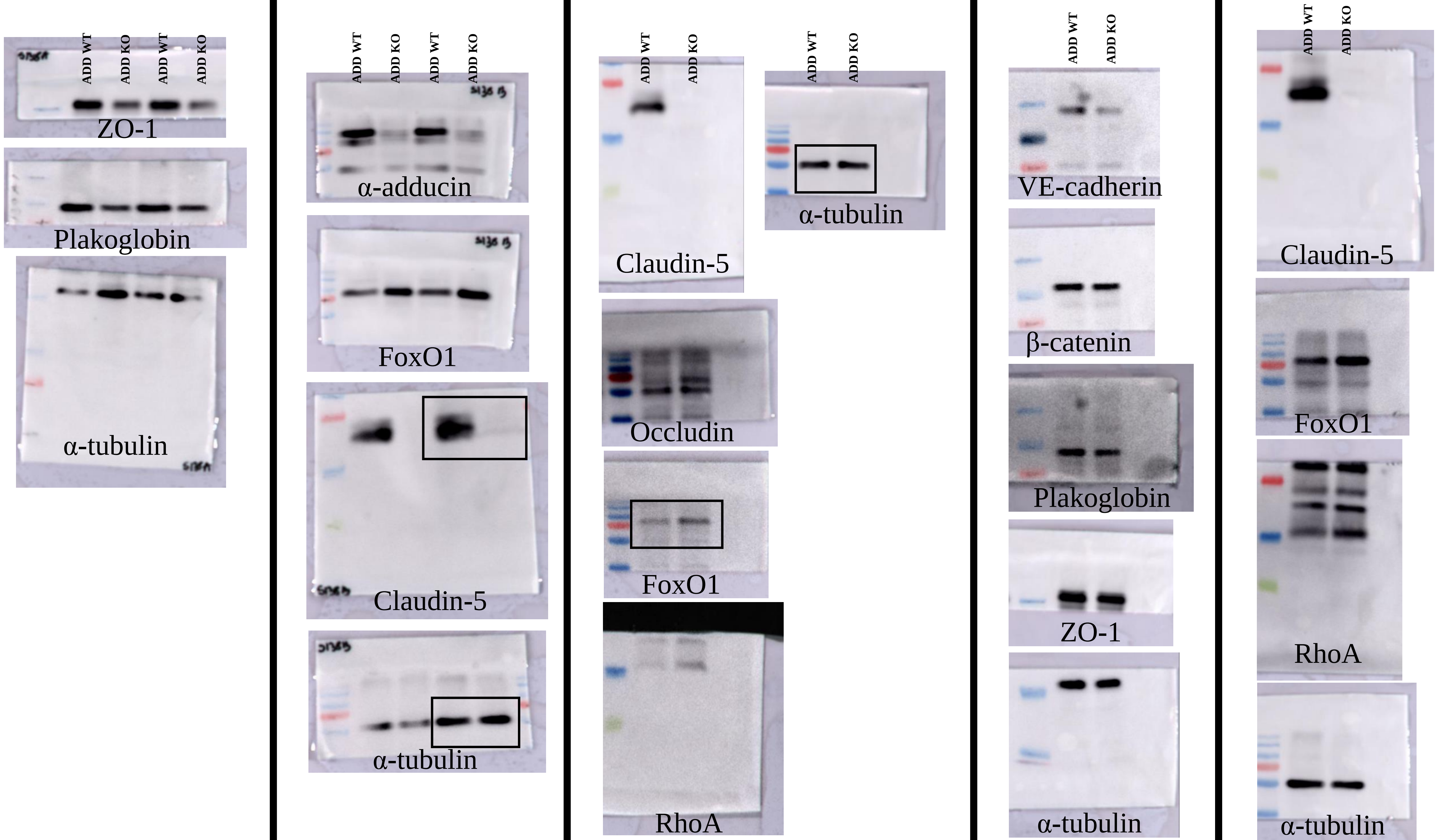

ADD WT
ADD KO
ADD WT
ADD KO
ZO-1
Plakoglobin
α-tubulin
ADD WT
ADD KO
ADD WT
ADD KO
α-adducin
FoxO1
Claudin-5
α-tubulin
ADD WT
ADD KO
Claudin-5
ADD WT
ADD KO
α-tubulin
Occludin
FoxO1
RhoA
ADD WT
ADD KO
VE-cadherin
β-catenin
Plakoglobin
ZO-1
α-tubulin
ADD WT
ADD KO
Claudin-5
FoxO1
RhoA
α-tubulin

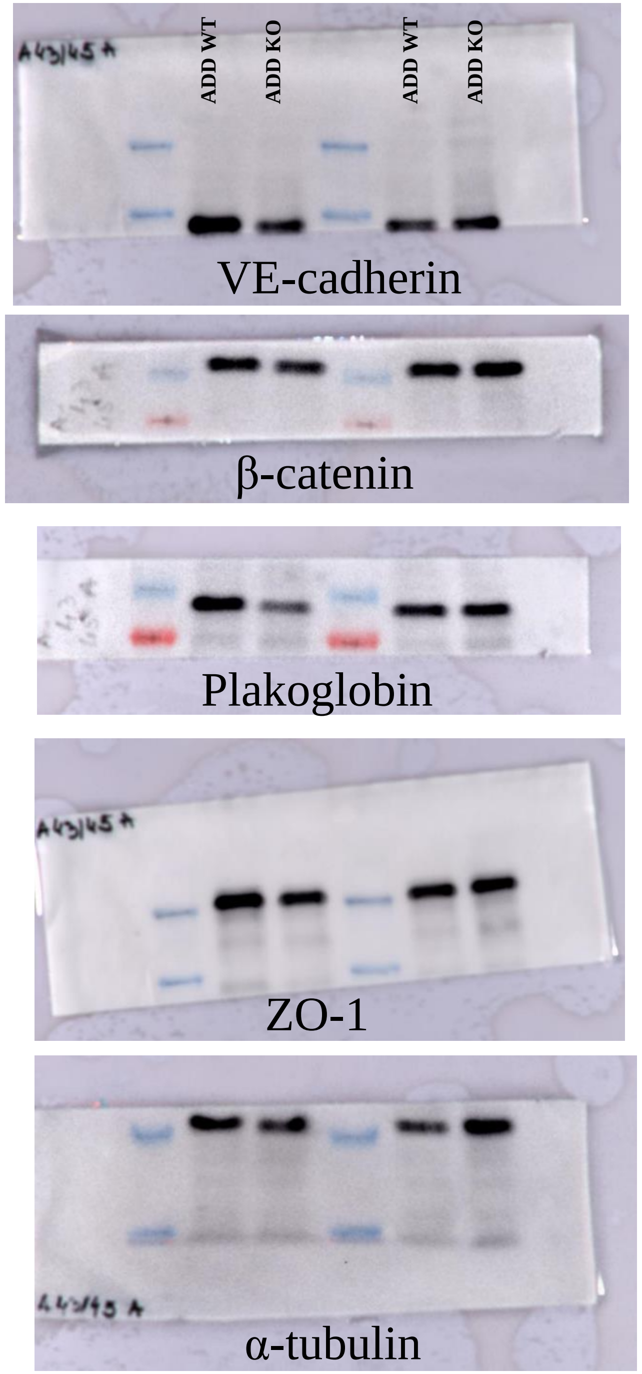

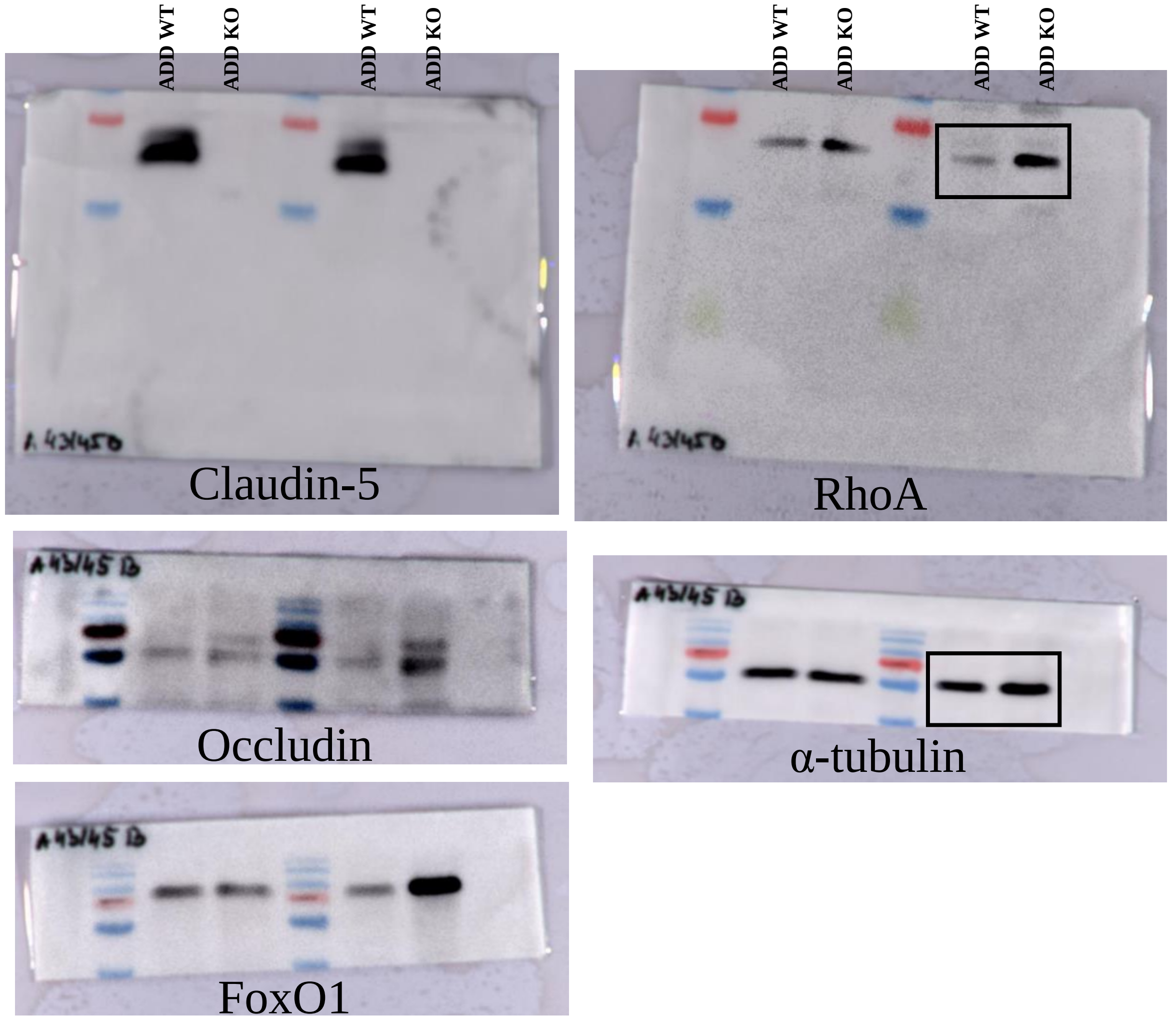

Each side from the molecular weight marker loaded in the middle is an independent N

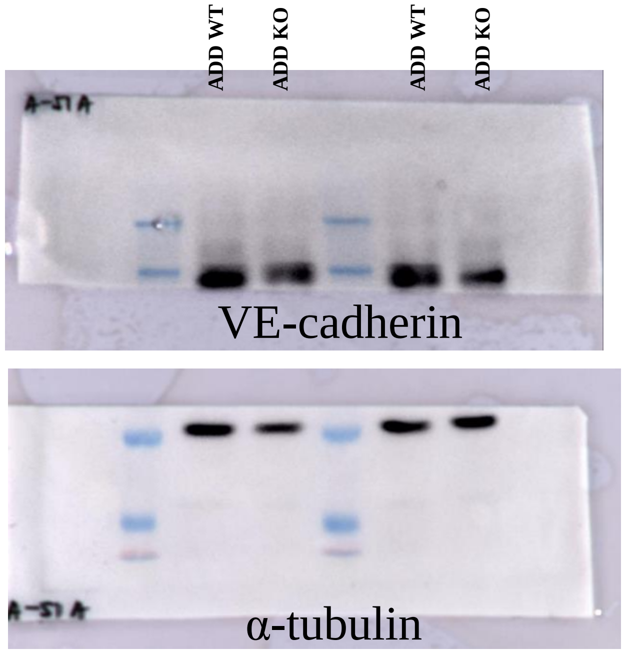

Each side from the molecular weight marker loaded in the middle is an independent N

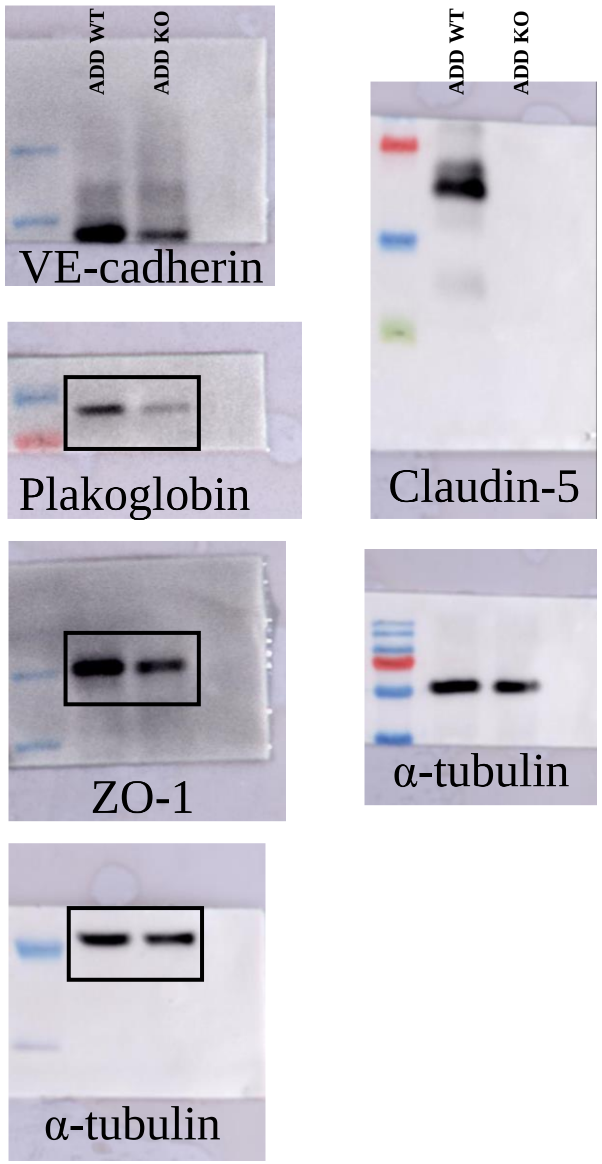
ADD WT
ADD KO
VE-cadherin
Plakoglobin
ZO-1
α-tubulin
ADD WT
ADD KO
Claudin-5
α-tubulin

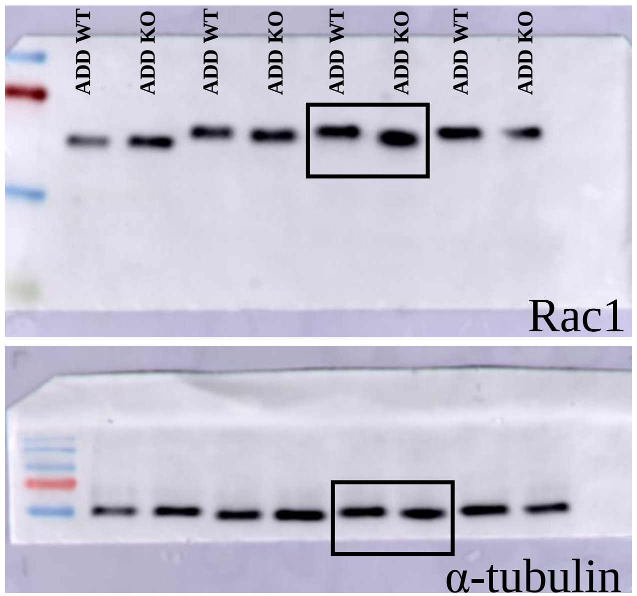
ADD WT
ADD KO
ADD WT
ADD KO
ADD WT
ADD KO
ADD WT
ADD KO
Rac1
α-tubulin

Original Western blot gel images for IPs

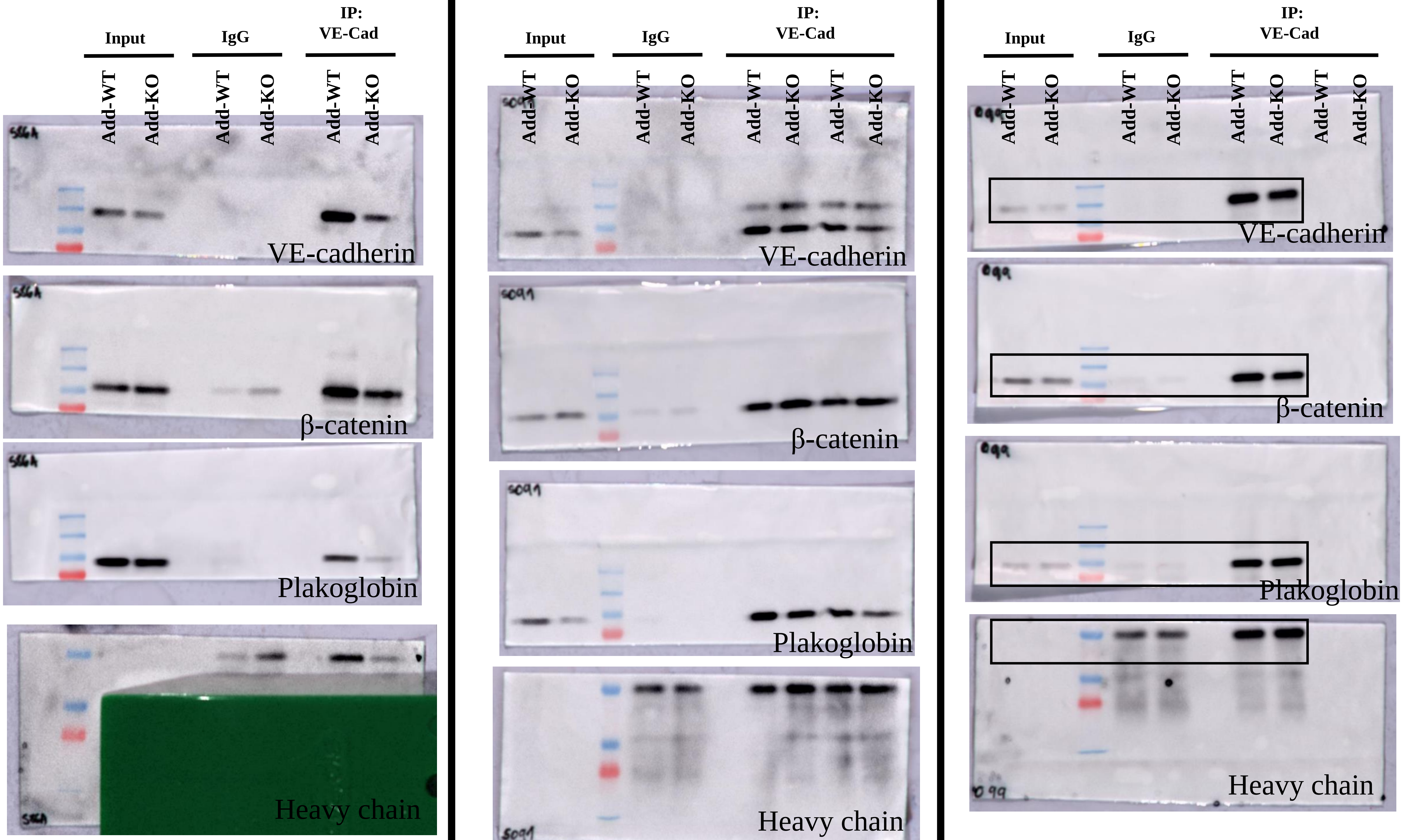
Input
IgG
IP: VE-Cad
Add-WT
Add-KO
Add-WT
Add-KO
Add-WT
Add-KO
VE-cadherin
β-catenin
Plakoglobin
Heavy chain
Input
IgG
IP: VE-Cad
Add-WT
Add-KO
Add-WT
Add-KO
Add-WT
Add-KO
Add-WT
Add-KO
VE-cadherin
β-catenin
Plakoglobin
Heavy chain
Input
IgG
IP: VE-Cad
Add-WT
Add-KO
Add-WT
Add-KO
Add-WT
Add-KO
Add-WT
Add-KO
VE-cadherin
β-catenin
Plakoglobin
Heavy chain

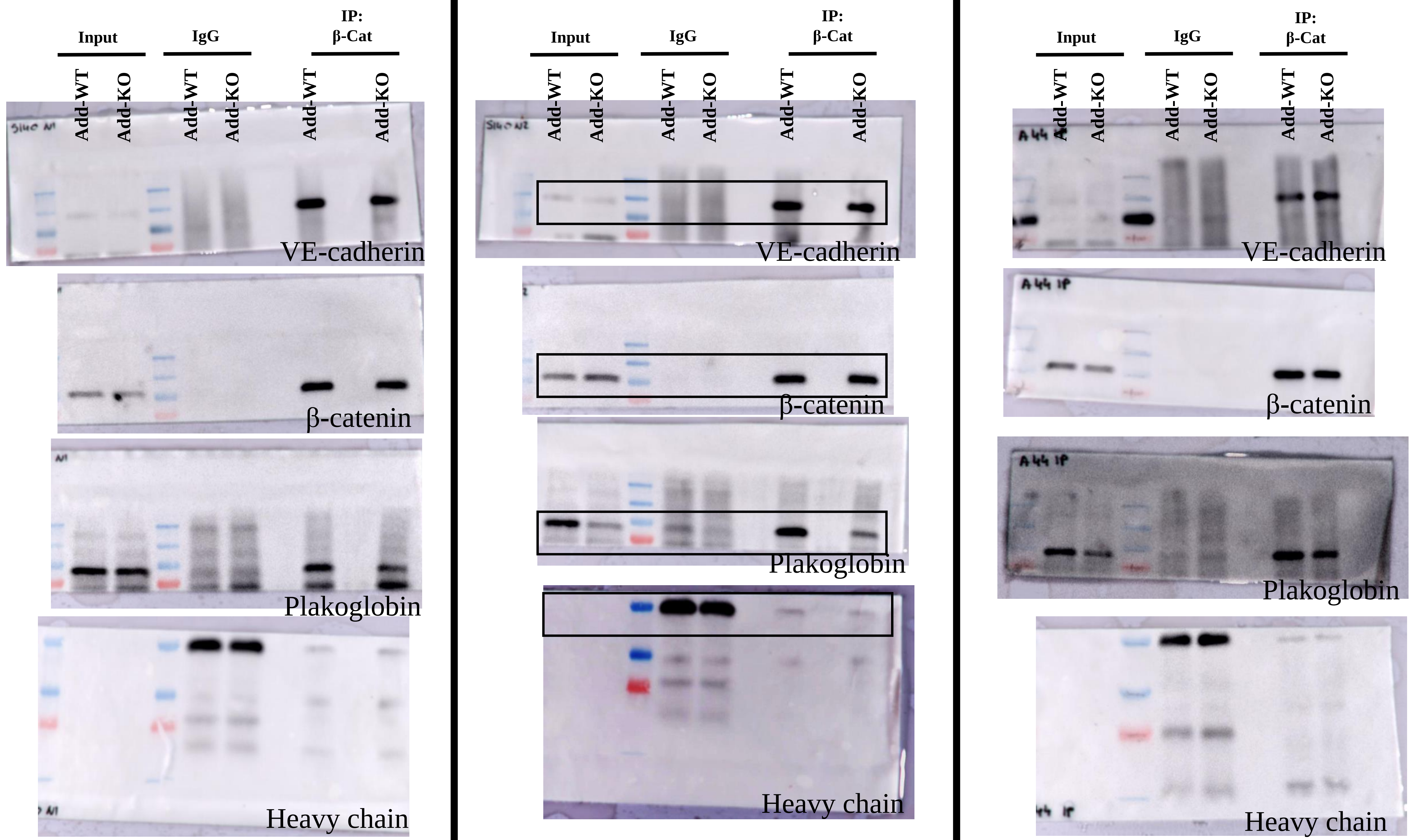
Input
IgG
IP:
β-Cat
Add-WT
Add-KO
Add-WT
Add-KO
Add-WT
Add-KO
VE-cadherin
β-catenin
Plakoglobin
Heavy chain
Input
IgG
IP:
β-Cat
Add-WT
Add-KO
Add-WT
Add-KO
Add-WT
Add-KO
VE-cadherin
β-catenin
Plakoglobin
Heavy chain
Input
IgG
IP:
β-Cat
Add-WT
Add-KO
Add-WT
Add-KO
Add-WT
Add-KO
VE-cadherin
β-catenin
Plakoglobin
Heavy chain

Original Western blot gel images for DMSO and F/R treatments

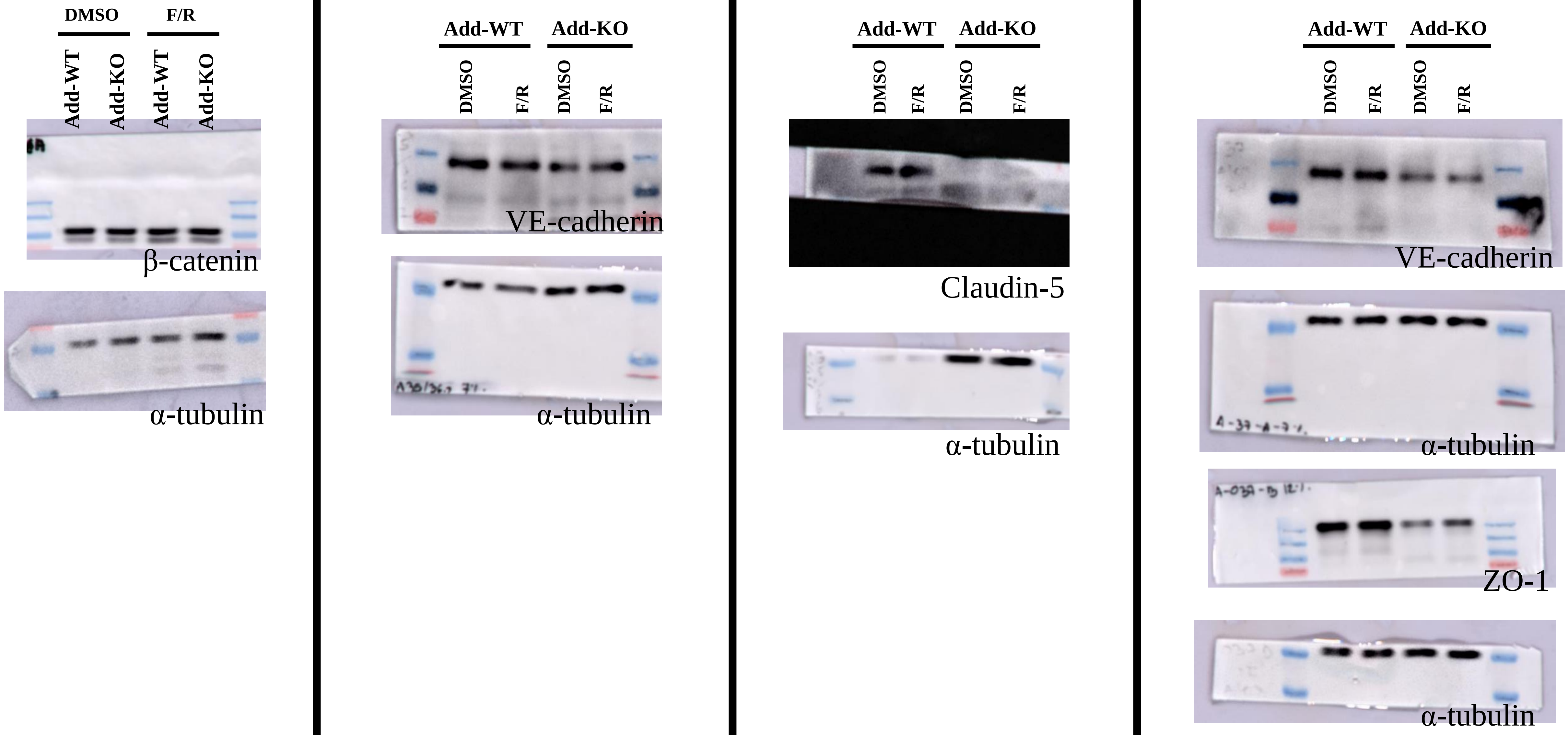
DMSO
F/R
Add-WT
Add-KO
Add-WT
Add-KO
β-catenin
α-tubulin
Add-WT
Add-KO
DMSO
F/R
DMSO
F/R
VE-cadherin
α-tubulin
Add-WT
Add-KO
DMSO
F/R
DMSO
F/R
Claudin-5
α-tubulin
Add-WT
Add-KO
DMSO
F/R
DMSO
F/R
VE-cadherin
α-tubulin
ZO-1
α-tubulin

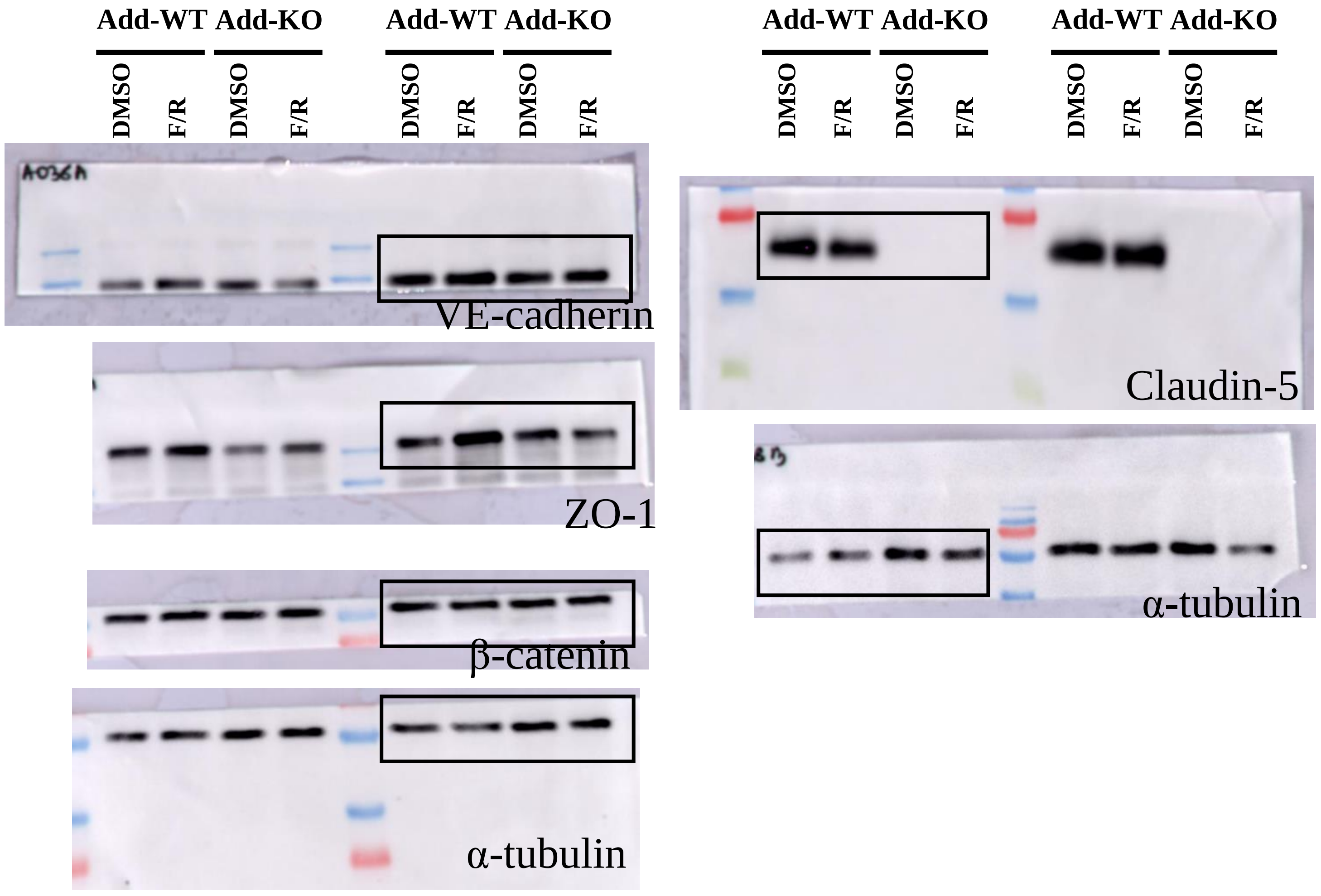

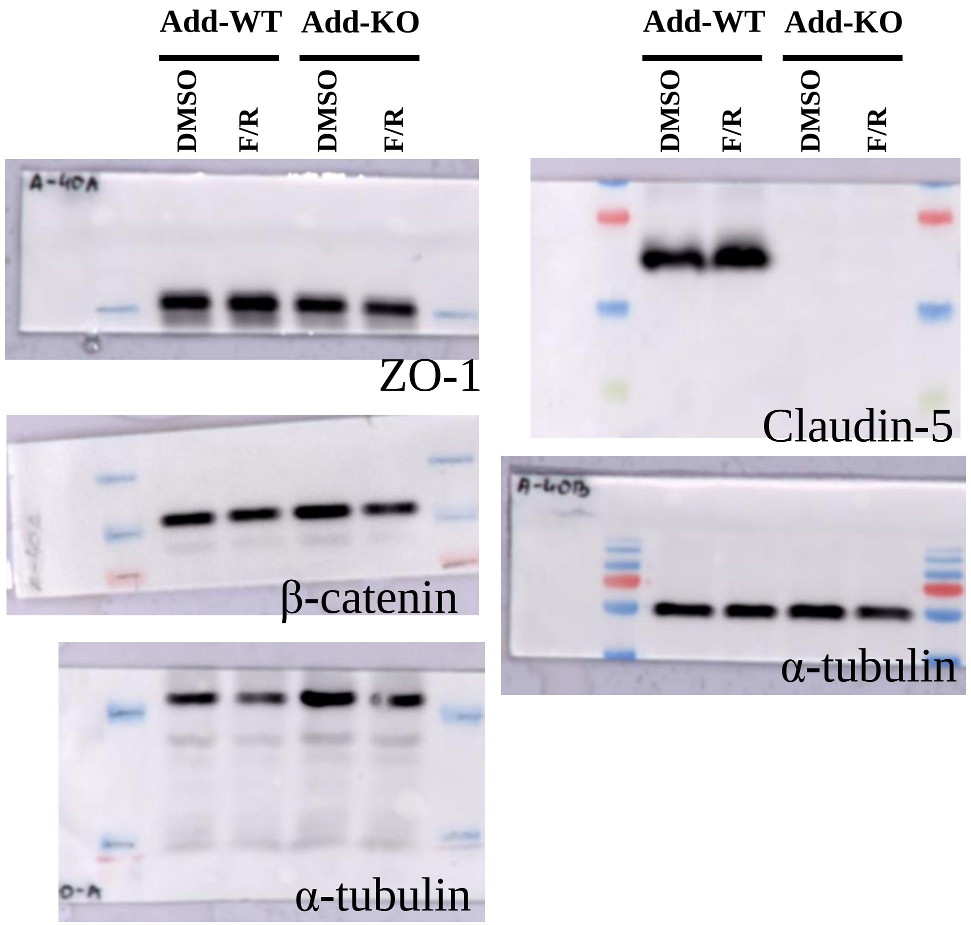

Each side from the molecular weight marker loaded in the middle is an independent N

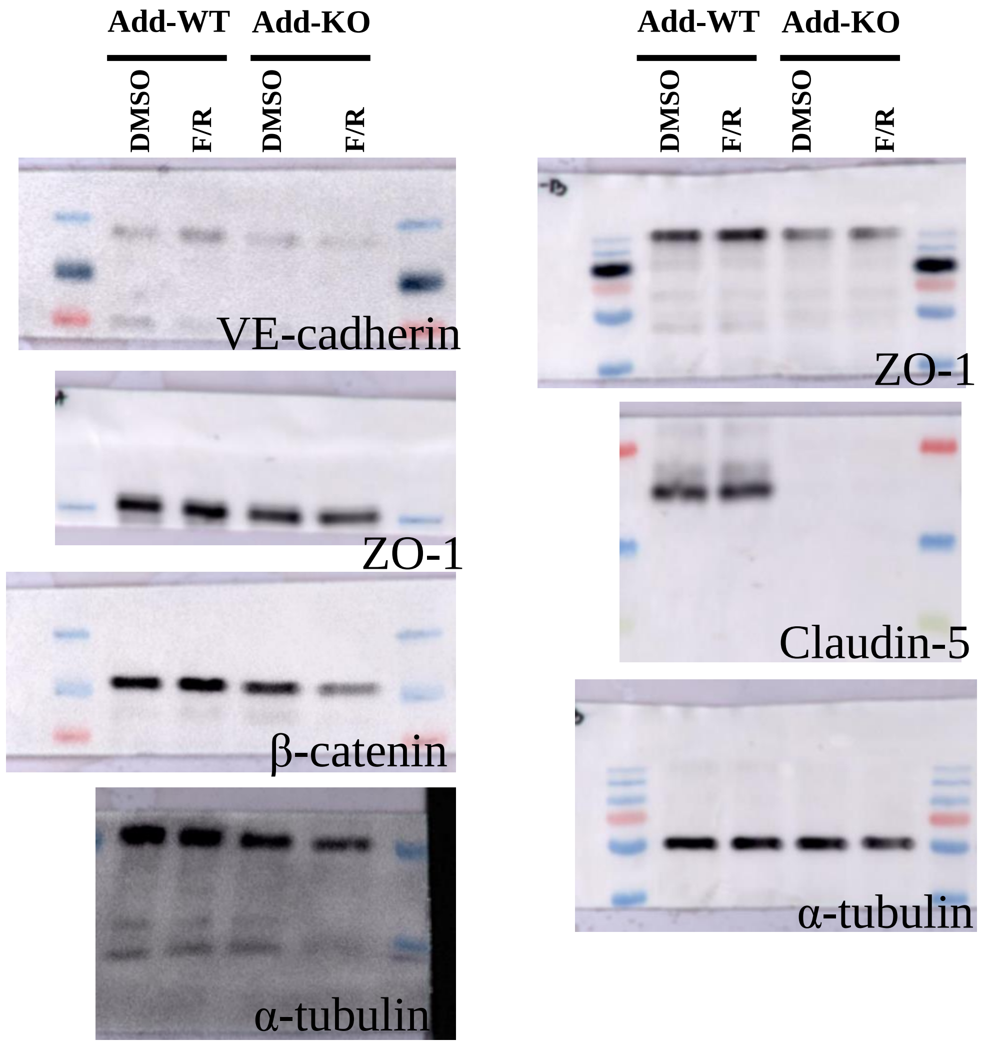

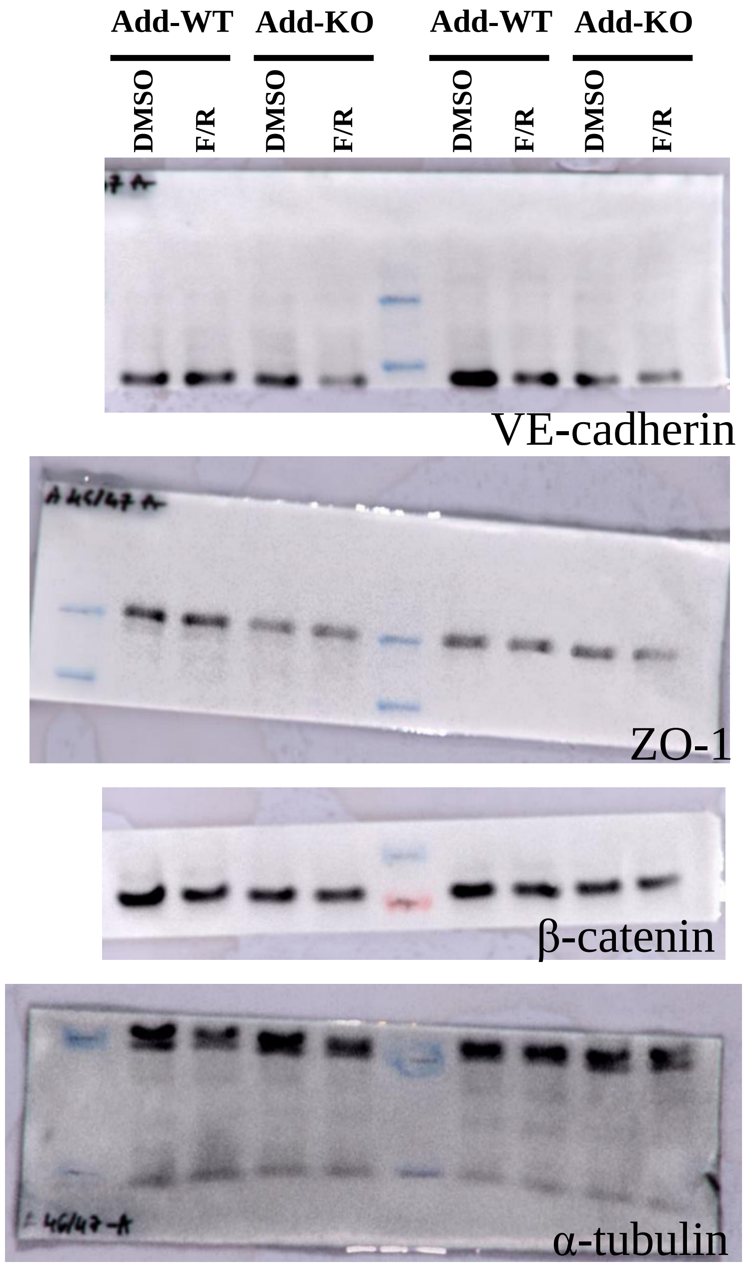

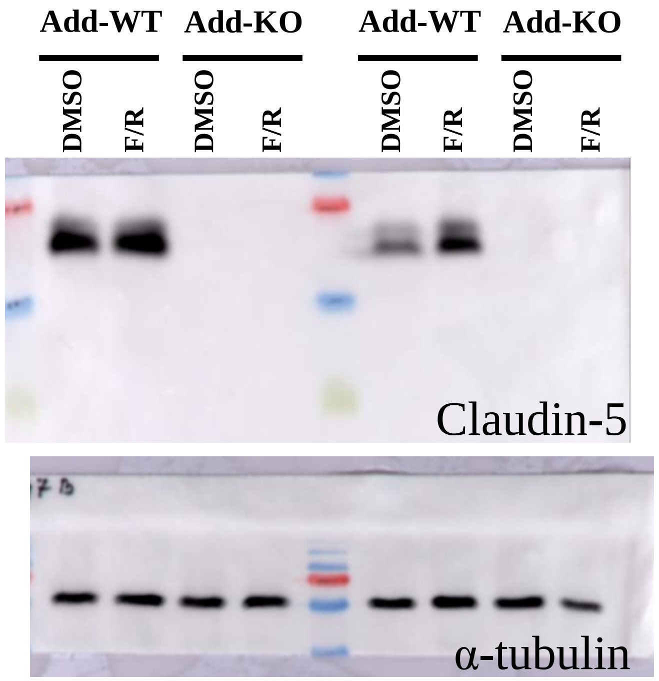

Each side from the molecular weight marker loaded in the middle is an independent N

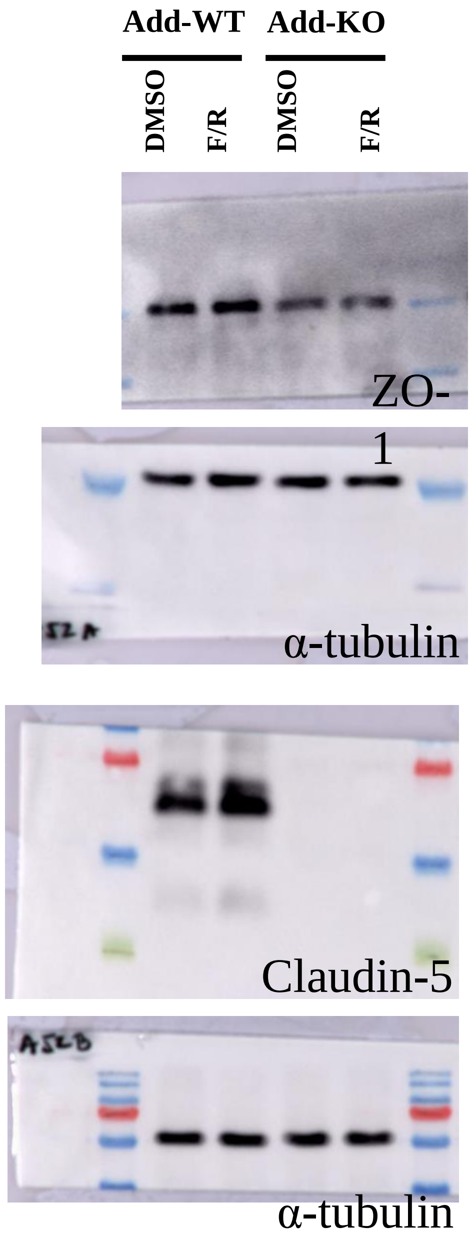

Add-WT
Add-KO
DMSO
F/R
DMSO
F/R
ZO-1
α-tubulin
Claudin-5
α-tubulin

Original Western blot gel images for Calcium Switch

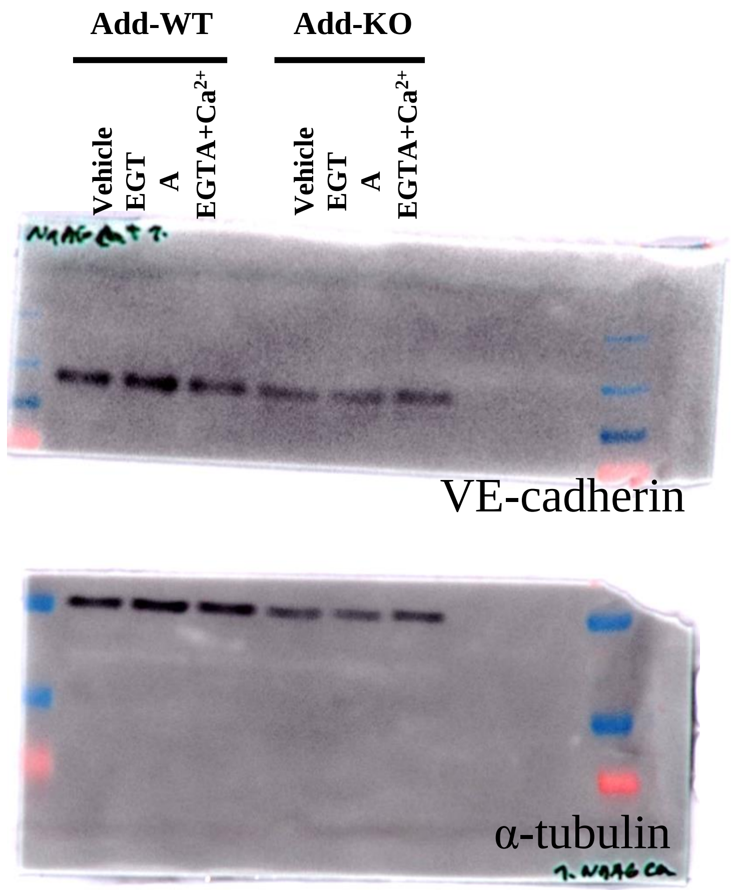

Add-WT
Add-KO
Vehicle
EGT
A
EGTA+Ca2+
Vehicle
EGT
A
EGTA+Ca2+
VE-cadherin
α-tubulin

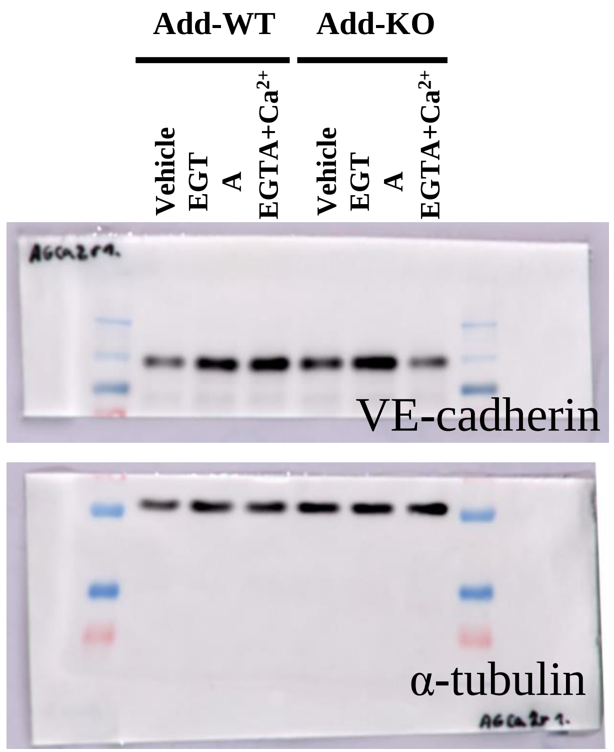

Add-WT
Add-KO
Vehicle
EGT
A
EGTA+Ca2+
Vehicle
EGT
A
EGTA+Ca2+
VE-cadherin
α-tubulin

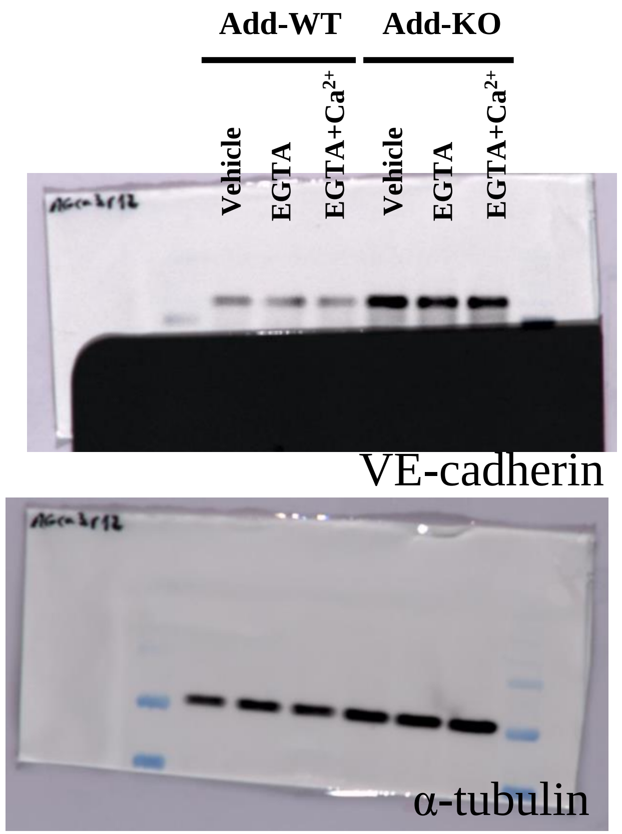

Add-WT
Add-KO
Vehicle
EGTA
EGTA+Ca2+
Vehicle
EGTA
EGTA+Ca2+
VE-cadherin
α-tubulin

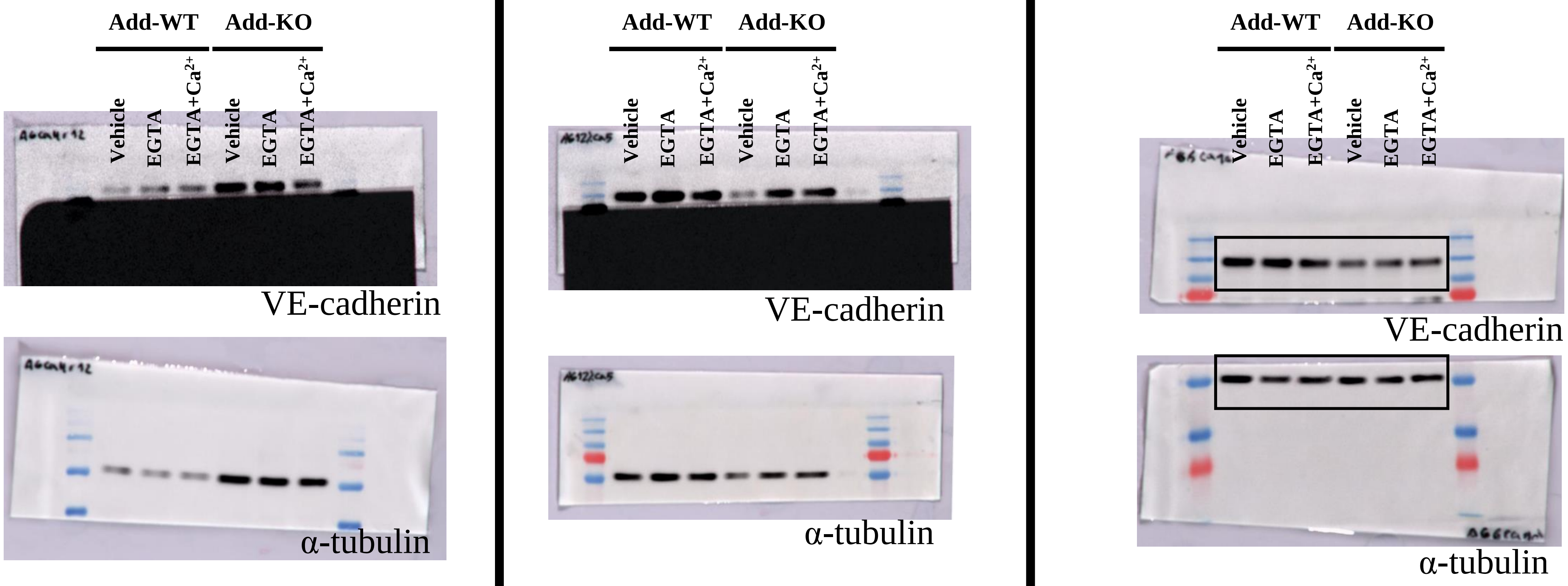
Add-WT
Add-KO
Vehicle
EGTA
EGTA+Ca^{2+}
Vehicle
EGTA
EGTA+Ca^{2+}
VE-cadherin
α-tubulin
Add-WT
Add-KO
Vehicle
EGTA
EGTA+Ca^{2+}
Vehicle
EGTA
EGTA+Ca^{2+}
VE-cadherin
α-tubulin
Add-WT
Add-KO
Vehicle
EGTA
EGTA+Ca^{2+}
Vehicle
EGTA
EGTA+Ca^{2+}
VE-cadherin
α-tubulin

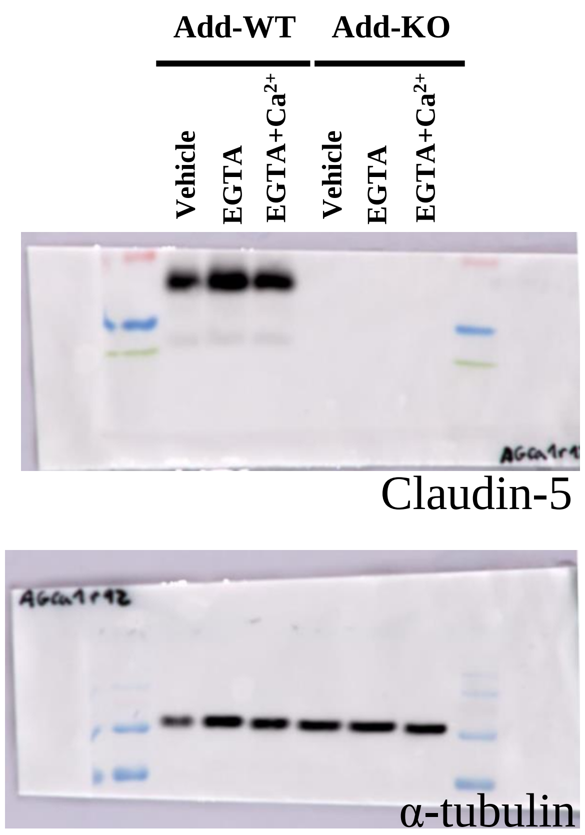

Add-WT
Add-KO
Vehicle
EGTA
EGTA+Ca2+
Vehicle
EGTA
EGTA+Ca2+
Claudin-5
α-tubulin

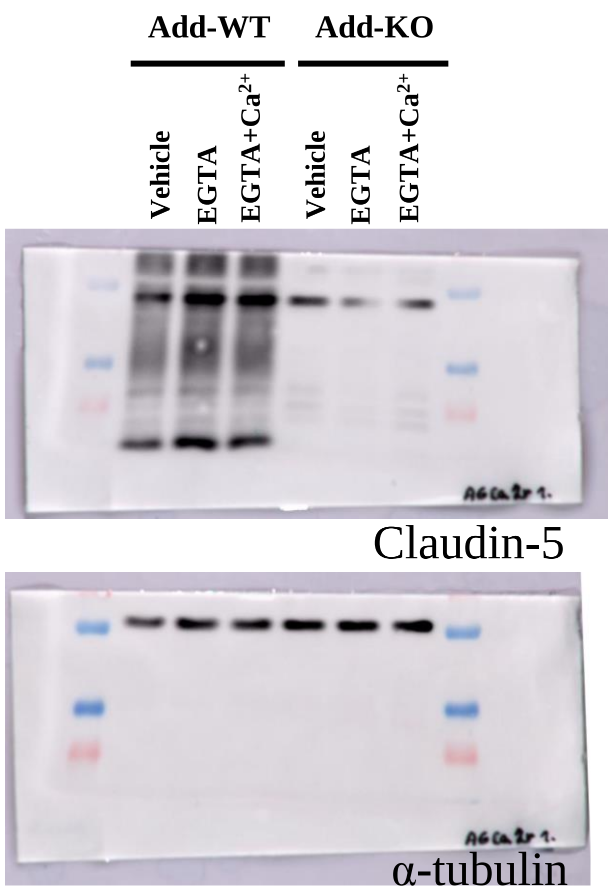

Add-WT
Add-KO
Vehicle
EGTA
EGTA+Ca2+
Vehicle
EGTA
EGTA+Ca2+
Claudin-5
α-tubulin

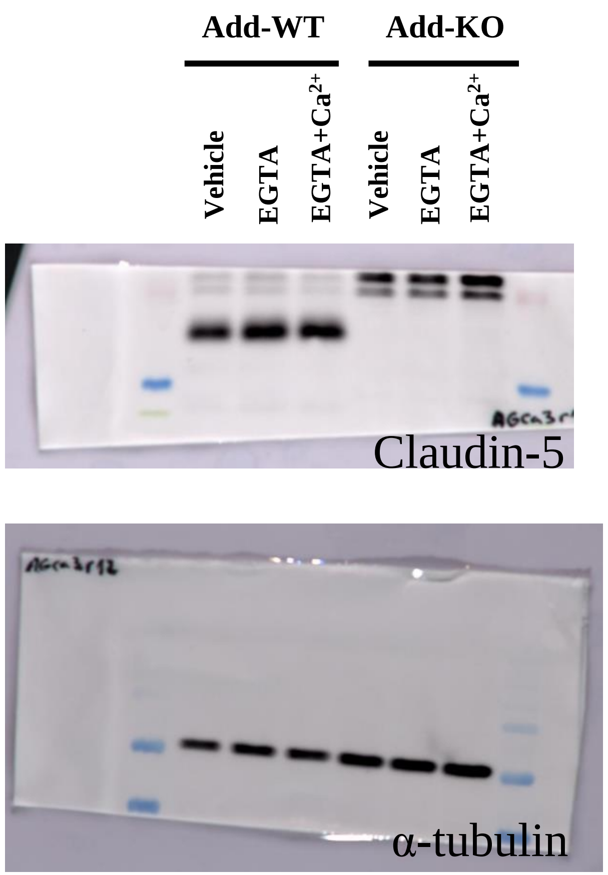

Add-WT
Add-KO
Vehicle
EGTA
EGTA+Ca2+
Vehicle
EGTA
EGTA+Ca2+
Claudin-5
α-tubulin

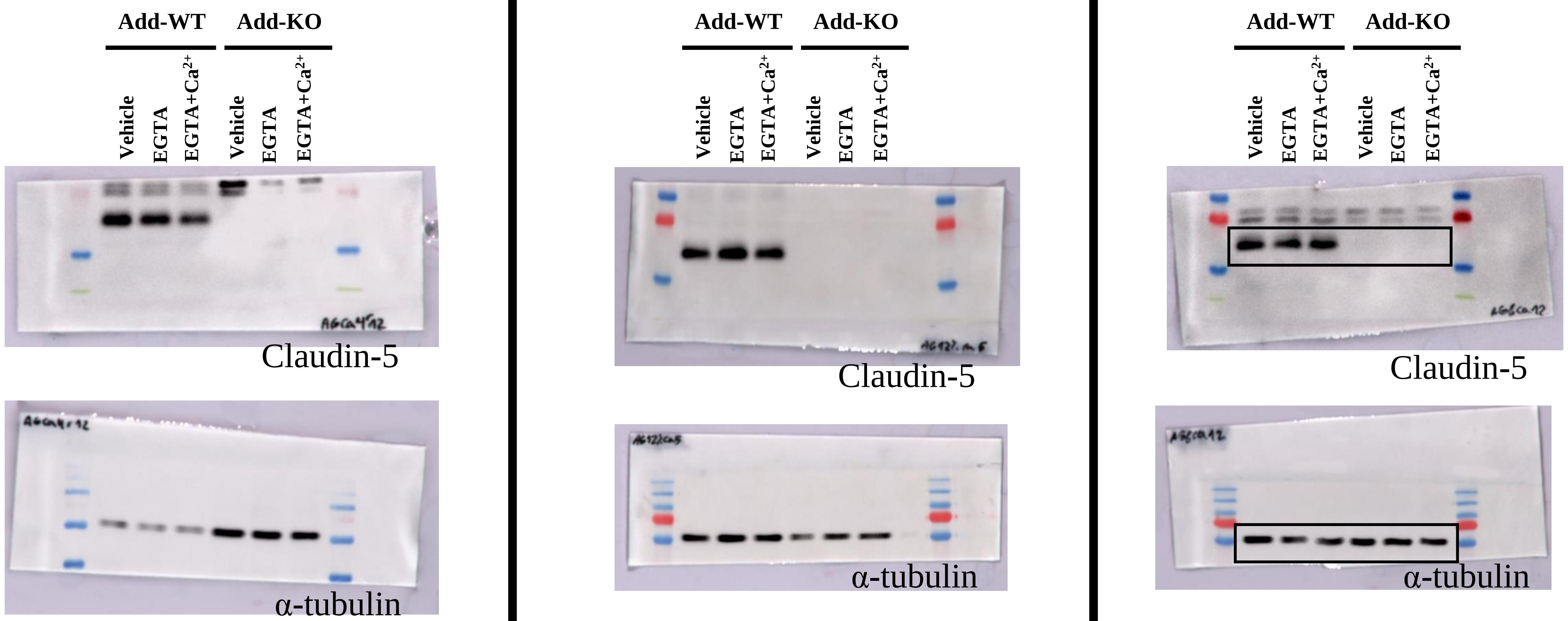

Add-WT
Add-KO
Vehicle
EGTA
EGTA+Ca^{2+}
Vehicle
EGTA
EGTA+Ca^{2+}
Claudin-5
α-tubulin
Add-WT
Add-KO
Vehicle
EGTA
EGTA+Ca^{2+}
Vehicle
EGTA
EGTA+Ca^{2+}
Claudin-5
α-tubulin
Add-WT
Add-KO
Vehicle
EGTA
EGTA+Ca^{2+}
Vehicle
EGTA
EGTA+Ca^{2+}
Claudin-5
α-tubulin
